# Transcriptional regulation of hormone signalling genes in black pepper in response to *Phytophthora capsici*

**DOI:** 10.1186/s12864-024-10802-4

**Published:** 2024-09-30

**Authors:** Chidambareswaren Mahadevan, K. Mohamed Shafi, B Nagarathnam, Manjula Sakuntala, Ramanathan Sowdhamini

**Affiliations:** 1 Plant Disease Biology and Biotechnology, Rajiv Gandhi Centre for Biotechnology, Thycaud P.O, Thiruvananthapuram, 695014 Kerala India; 2 National Centre for Biological Sciences (TIFR), GKVK Campus, Bangalore, 560065 Karnataka India; 3 Department of Biology, University of Oxford, Oxford, England

**Keywords:** Plant innate immunity, *Piper nigrum*, *Phytophthora capsici*, Transcriptomics, Hormone signalling, Gene expression

## Abstract

**Introduction:**

Black pepper (*Piper nigrum* L.) is a non-model spice crop of significant agricultural and biological importance. The ‘quick wilt’ disease caused by the oomycete *Phytophthora capsici* is a major threat, leading to substantial crop loss. The molecular mechanisms governing the plant immune responses to this pathogen remain unclear. This study employs RNA sequencing and transcriptome analysis to explore the defense mechanisms of *P. nigrum* against *P. capsici*.

**Results:**

Two-month-old *P. nigrum* plantlets were subjected to infection with *P. capsici*, and leaf samples were collected at 6- and 12-hours post-inoculation. RNA was extracted, sequenced, and the resulting data were processed and assembled. Differential gene expression analysis was conducted to identify genes responding to the infection. Additionally, the study investigated the involvement of Salicylic acid (SA), Jasmonic acid (JA), and Ethylene (ET) signalling pathways. Our transcriptome assembly comprised 64,667 transcripts with 96.7% completeness, providing valuable insights into the *P. nigrum* transcriptome. Annotation of these transcripts identified functional categories and domains, provided details on molecular processes. Gene expression analysis identified 4,714 transcripts at 6 h post-infection (hpi) and 9,416 at 12 hpi as differentially expressed, revealing dynamic regulation of immune-related genes. Furthermore, the study investigated key genes involved in biosynthesis pathways of Salicylic acid, Jasmonic acid, and Ethylene signalling. Notably, we found differential regulation of critical genes associated with these pathways while comparing data before and after infection, thereby shedding light on their roles in defense mechanism in *P. nigrum* defense.

**Conclusions:**

This comprehensive transcriptome analysis of *P. nigrum* response to *P. capsici* attack provides valuable insights into the plant defense mechanisms. The dynamic regulation of innate immunity and the involvement of key signalling pathways highlight the complexity of the plant-pathogen interaction. This study contributes to our understanding of plant immunity and offers potential strategies for enhancing *P. nigrum* resistance to this harmful pathogen.

**Supplementary Information:**

The online version contains supplementary material available at 10.1186/s12864-024-10802-4.

## Introduction

*Piper nigrum L*., commonly known as black pepper, is a significant spice crop both in terms of agronomy and biology, despite not being a model plant species. One of the primary challenges faced in *P. nigrum* cultivation is the occurrence of ‘quick wilt,’ a disease caused by a compatible interaction with the hemibiotrophic oomycete, *Phytophthora capsici* [1]. This pathogen alone is responsible for causing over 30% of vine mortality and crop losses in *P. nigrum* [2]. The molecular mechanisms underlying Pattern-Triggered Immunity (PTI) or Effector-Triggered Immunity (ETI) responses in *P. nigrum* have remained largely undiscovered [3].

The significance of Salicylic acid (SA), Jasmonic acid (JA), and Ethylene (ET) in plant defense mechanisms is well-established and essential for a plant ability to combat pathogens and adapt to environmental challenges. SA serves as a crucial signalling molecule in defense against biotrophic pathogens, inducing the expression of pathogenesis-related (PR) genes that lead to the production of antimicrobial compounds [4]. In contrast, JA and its associated pathway primarily defend against necrotrophic pathogens and herbivores by triggering the synthesis of protease inhibitors, defensive proteins, and secondary metabolites [5]. Ethylene, often working synergistically with JA, plays a broad role in defense responses, including the regulation of senescence, cell wall modification, and the induction of defense-related genes [6]. The intricate crosstalk between these signalling pathways allows plants to fine-tune their responses to specific threats and environmental conditions, ultimately enhancing their ability to withstand diverse challenges.

Understanding the molecular basis of compatible interactions between plants and pathogenic oomycetes remains a significant challenge in the study of plant-pathogen interactions [7]. RNA sequencing combined with *de novo* transcriptome assembly has been successfully applied to non-model plants, including *P. nigrum*, for molecular insights [3, 8]. Our previous work provided valuable molecular insights into *P. nigrum* defense responses against *P. capsici*, demonstrating the hemibiotrophic early pathogenicity and the host susceptibility within 24 h post-infection [9]. This led us to hypothesize that innate immunity might be a crucial mechanism manipulated by *P. capsici*, facilitating its early compatible interaction with *P. nigrum*. To explore the role of innate immunity, we conducted an extensive study involving comparative foliar transcriptomics of *P. nigrum* during *P. capsici* infection.

This study presents a comprehensive analysis of *P. nigrum* transcriptome data and identifies critical immune components involved in disease resistance against various pathogens. Comparative transcriptome analysis of *P. nigrum* leaves from control and infected samples revealed several differentially expressed genes. Our findings highlight the conservation of molecular immune components in *P. nigrum* and demonstrating dynamic regulation in response to *P. capsici* challenge. Additionally, we report the identification, in silico analysis and differential expression analysis of transcripts to provide further insights into the molecular changes accompanying the hemi-biotrophic pathogenicity of *P. capsici* in *P. nigrum*. The findings of this study can be developed further for application in protecting *P. nigrum* by ‘priming’ which we have established as a potential crop protection strategy in this spice crop [10].

## Results and discussion

In this study, we used the Panniyur I variety of *P. nigrum*, which is known to be susceptible to *P. capsici*. This cultivar is a widely grown variety and is relevant for exploring defense strategies such as defense priming. Our protocols were optimized using this variety to ensure consistency and applicability of the findings. *P. capsici* is a biotrophic pathogen that initiates infection and begins to show microscopically visible growth on the infected tissue by 6 hpi. The infection progresses with hyphal proliferation, and by 24 hpi, clear signs of necrosis in host tissue are visible, indicating a transition to the necrotrophic phase of the pathogen lifestyle. We selected 6 and 12 hpi to represent the biotrophic phase and the early stage of the necrotrophic phase, respectively. The later time point of 24 hpi was avoided for transcript analysis as the significant host cell death and necrosis at this stage could interfere with sensitive assays, such as HR, ROS generation, and fluorescence assays.

### *De novo* assembly and quality assessment of *P. nigrum* transcriptome

To investigate the molecular elements involved in immunity within *P. nigrum*, our study focused to establish a comparative transcriptome dataset with differentially expressed genes. This was accomplished by utilizing RNA-seq data acquired from leaves that were exposed to *P. capsici*. Our preliminary data analysis resulted in the generation of approximately 216 million paired end reads (Additional File 1). After initial pre-processing steps, we obtained high-quality data, with over 87% of the bases exhibiting an average base quality Q > 30 and a mean GC content of 43.81%. By utilizing a high-quality genome of *P. nigrum* as guidance, we generated a transcriptome assembly that comprised a total of 64,667 transcripts (Table 1). The transcript assembly exhibited a percent GC content of 44.04, and an N50 value of 2507 was observed, indicating the contiguity of the assembly. The cumulative length of the assembly was 129.43 Mb, with 50,696 contigs surpassing a length of 1000 base pairs. The largest contig identified in the assembly measured 27,925 base pairs. Notably, 84% of the reads were successfully mapped back to the assembly, indicating the comprehensive nature and accuracy of the transcriptome assembly. The assembly was further tested for completeness using BUSCO against *Viridiplantae* database and found a good 96.7% completeness for the transcripts.

**Table 1:** Reference based transcriptome assembly statistics of *P. Nigrum*. The percentage value of BUSCO completeness shown in C: complete; S: complete and single-copy; D: complete and duplicated; F: fragmented; M: missing; N: number of genes

Assembly statistics	
**Transcripts**	
Number of sequences	64,667
Total length	129,429,265 bp
Longest sequence	27,925 bp
Shortest sequence	200 bp
N50	2507 bp
GC content	44.40%
BUSCO (N:425)	C:96.7% [S:32.7%, D:64.0%], F:0.7%, M:2.6%
**Unigenes**	
Number of sequences	74,607
Total length	25,127,195 aa
Longest sequence	5371 aa
Shortest sequence	86 aa
BUSCO (N:425)	C:87.8% [S:44.7%, D:43.1%], F:8.9%, M:3.3%

### Functional annotation of *P. nigrum* transcriptome

The transcriptome assembly *of P. nigrum* predicted 74,607 ORFs (unigenes) using TransDecoder program. We performed comprehensive homology searches against the Uniprot-*Viridiplantae* database for both transcripts and unigenes to functionally annotate the transcriptome. We used programs such as BLASTP for unigenes and BLASTX for transcripts, and 86.02% and 69.71% of them were effectively annotated, respectively. Notably, majority of these homologues had been found in cinnamon and lotus species, providing further insight into potential evolutionary relationships.

The functional analysis of the homologues provided insights into the diverse roles of the identified proteins. We observed an abundance protein kinase domain-containing, ubiquitin-related, and pentatricopeptide repeat (PPR) proteins. Protein kinases are critical in signal transduction pathways, including those involved in stress and immune responses. Their prevalence in our dataset highlights the potential importance of phosphorylation events in regulating the immune response to *P. capsici*. Ubiquitin-related proteins are involved in the ubiquitination process, which tags proteins for degradation or modification, thus playing a role in regulating protein turnover and cellular responses to stress. The presence of these proteins suggests that ubiquitin-mediated processes are significant in modulating the plant response to pathogen attack. PPR proteins, which are involved in RNA processing and regulation, were also notably abundant. These proteins play roles in post-transcriptional regulation, which is crucial for fine-tuning gene expression in response to environmental stimuli. The high number of PPR proteins may reflect the need for tight control over gene expression during infection. Among the annotated proteins, we found a substantial presence of RNA-binding proteins, such as those containing the RNA Recognition Motif (RRM). RNA-binding proteins are essential for various aspects of RNA metabolism, including splicing, stabilization, and transport. Their abundance suggests a significant role in regulating gene expression and processing of transcripts in response to pathogen infection. We also identified several disease resistance proteins, including those with Leucine-Rich Repeat (LRR) domains. These proteins are known for their role in recognizing pathogen-derived molecules and initiating defense responses. The presence of LRR domain-containing proteins indicates that *P. nigrum* employs these receptors as part of its immune system to detect and respond to pathogen presence. Furthermore, we used HMMScan to predict domains against the Pfam database, and 79.89% of the unigenes were allocated domains. PPR and LRR domains were found to be particularly prevalent, providing vital insights into the molecular machinery of the *P. nigrum* transcriptome. The identification of PPR domains aligns with our earlier observations and underscores the importance of RNA regulation in the immune response. The abundance of LRR domains further supports the hypothesis that *P. nigrum* relies on receptor-like proteins to detect and respond to pathogen challenges. MSTRG.20872.1 (Pentatricopeptide repeat containing protein) with PPR domains and MSTRG.13016.1 (Disease resistance protein) with LRR domains were the most abundant transcripts after infection. The upregulation of these transcripts during infection highlights their potential importance in the plant defense mechanism. The PPR-containing protein might play a role in regulating the expression of genes involved in stress response, while the LRR-containing protein could function as a pathogen receptor, initiating defense signaling pathways.

### Comparative transcriptomics reveals the differentially expressed genes

**Fig. 1:**
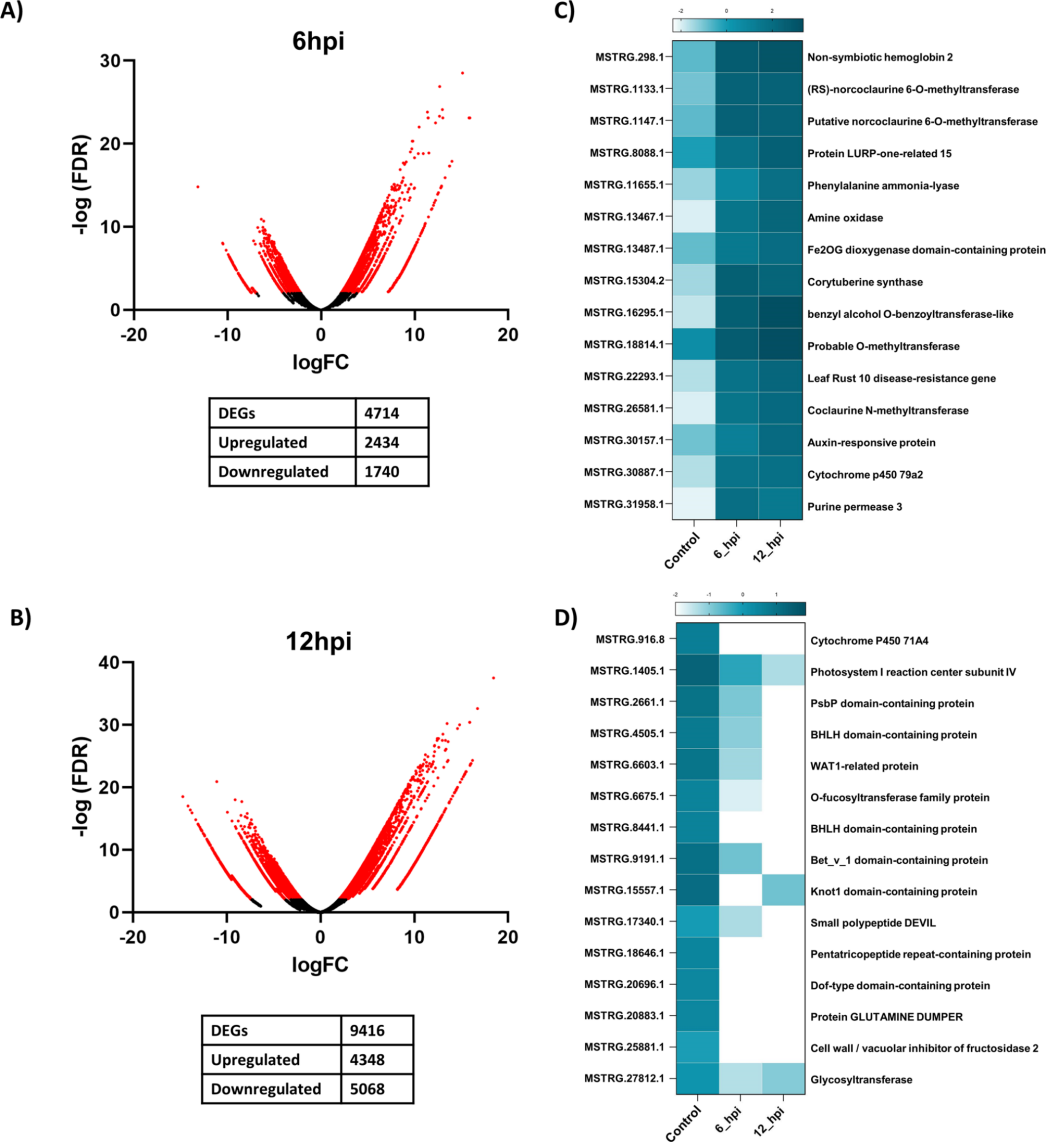
Comparative gene expression data derived for three samples (control, 6 hpi and 12 hpi) (**A**) Volcano plot for control vs. 6 hpi (**B**) Volcano plot for control vs. 12 hpi. (**C**) heatmaps showing top 15 differentially upregulated transcripts and (D) top 15 differentially downregulated transcripts from *P. nigrum*. Genes with a log_2_ fold change of ± 1.5 and FDR < 0.05 were considered to be differentially expressed genes

To elucidate the transcriptional changes related to innate immunity in *P. nigrum* during the early biotrophic colonization of *P. capsici*, we performed a comparative transcriptomics approach that generated differential gene expression between control and infected samples, namely *P. capsici* infection, sampled after 6 hpi and 12 hpi. An analysis using edgeR, representing the logarithmic fold changes as a function of the mean of normalized counts revealed differentially expressed genes (DEGs). We found a total of 4,714 transcripts at 6 hpi and 9,416 transcripts at 12 hpi that showed significant differential expression compared to the control. These transcripts exhibited a log2 fold change of ± 1.5 in the TPM expression level and were statistically significant (FDR < 0.05). Among these DEGs, we observed 2,434 upregulated transcripts and 1,740 downregulated transcripts at 6 hpi respectively. At 12 hpi, there were 4,348 upregulated transcripts and 5,068 downregulated transcripts respectively. These DEGs likely represent the innate immune signature components of *P. nigrum*, indicating their involvement in perceiving and activating defense responses against *P. capsici* early in the infection process. Further, the hierarchical clustering of the DEGs across these conditions, along with the Volcano plots generated from edgeR analysis, clearly distinguished the coherence between the infected conditions (Fig. 1A **and B)**.

We analysed 15 top upregulated and downregulated transcripts from the differential expression analysis of control and infected (6 hpi and 12 hpi) samples (Fig. 1C **and D**, Additional File 2). We observed a significant upregulation of several methyltransferase genes in the infected samples, indicating their potential involvement in the response to the infection. Methylation is known to affect gene silencing and transcriptional regulation, which may be crucial in modulating the plant defense mechanisms [11]. Additionally, within the top upregulated transcripts, we identified several genes associated with defense responses, including a leaf rust resistance gene, an auxin response gene, and an amine oxidase gene. The leaf rust resistance gene may provide cross-reactivity in defense mechanisms, while auxin response genes are involved in regulating growth and stress responses. Amine oxidases are known to participate in the oxidative burst and pathogen defense [12, 13]. Conversely, among the downregulated transcripts, we observed the suppression of genes such as bHLH transcription factors, genes related to photosystem functions, and genes associated with cell wall processes. bHLH factors are involved in various cellular processes, including the regulation of gene expression in response to environmental stimuli. During pathogen attack, plants often redirect energy and resources towards defense and repair processes, leading to reduced photosynthetic activity and alterations in cell wall composition [14, 15]. These downregulated genes may indicate a downscaling of certain cellular processes during the infection.

Our Gene Ontology (GO) term enrichment analysis for both upregulated and downregulated genes at 6 and 12 hpi provided insights into the functional differences in the plant response to pathogen attack. The top 20 GO terms across three categories biological process, molecular function, and cellular component were examined (Additional File 3). We observed that upregulated genes at both 6 hpi and 12 hpi were predominantly associated with metabolic processes and catalytic activities. This suggests that the plant mounts a robust metabolic response early and maintains this response as the infection progresses, likely to fuel the energy-intensive processes involved in defense. Conversely, downregulated genes at both time points were primarily linked to cellular processes and DNA-binding activities. The suppression of these functions may reflect a strategic reallocation of resources, prioritizing defense mechanisms over regular cellular maintenance and growth.

### Analysis on key hormone signalling pathways in defense response

Plants employ a complex defense mechanism to resist pathogenic agents. The SA, JA and ET mediated signalling pathways are recognized as an essential components of plant immune responses against pathogens. These signalling molecules play crucial roles in regulating plant defense pathways, enabling appropriate responses to various types of pathogens. SA initiates early defense-related gene expression in pathogen-infected plants, while JA induces late defense-related gene expression in pathogen-infected plants. JA is widely involved in regulating disease resistance against necrotrophic pathogens, while SA mediates broad-spectrum resistance against biotrophic and hemi biotrophic pathogens [16]. ET is another key player in plant defense, particularly in conjunction with SA and JA and it often more involved in defense against necrotrophic pathogens along with JA. The interplay between SA, JA, and ET signaling pathways is fundamental to the plant ability to mount a coordinated and effective defense response.

### Salicylic acid signalling pathway

In our study, we investigated three key enzymes within the SA signalling pathway: Non-expressor of pathogenesis-related genes 1 (NPR1), WRKY transcription factor 70 (WRKY70), and Pathogenesis-related protein 1 (PR1) [17], which play pivotal roles in the SA-mediated defense mechanism. NPR1, serving as the central orchestrator for the SA signalling pathway, and acts as a transcriptional co-activator alongside TGA transcription factors, promoting the expression of defense-related genes like PR1. In our analysis of the *P. nigrum* transcriptome, we identified two transcripts (MSTRG.29868.1 and MSTRG.29924.1) that contain the sequence signatures of NPR1. Interestingly, these NPR1-related transcripts were significantly upregulated at 6 hpi (Table 2, Additional File 4)., which suggests an early activation of SA signaling in response to *P. capsici* infection. This upregulation aligns with the known role of NPR1 in initiating defense responses. However, the observed decline in transcript levels at 12 hpi might indicate a downregulation phase, possibly due to the activation of feedback mechanisms or the transition to other signaling pathways as the infection progresses.

**Table 2:** Transcript expression of key enzymes involved in the SA, JA and ET hormone signalling pathways from three samples

Pathway genes	Transcript hits	TPM values		
		Control	6 hpi	12 hpi
**Salicylic acid**				
Non-expressor of pathogenesis-related genes 1 (NPR1)	MSTRG.29868.1	13.23	107.39	40.67
	MSTRG.29924.1	11.53	76.94	24.88
WRKY transcription factor 70 (WRKY70)	MSTRG.8862.1	20.64	93.07	48.83
Pathogenesis-related protein 1 (PR1)	MSTRG.17950.1	0.84	143.17	9.07
	MSTRG.18009.1	0.49	385.34	6.74
Glutaredoxin-C9 (GRX4)	MSTRG.5776.1	7.34	22.59	31.78
Transcription factor (TGA1)	MSTRG.1644.1	38.29	3.08	5.44
Transcription factor (TGA4)	MSTRG.4306.1	22.49	11.73	17.73
**Jasmonic acid**				
Coronatine Insensitive 1 (COI1)	MSTRG.25426.1	7.36	8.52	12.44
Jasmonate ZIM domain protein 1 (JAZ1)	MSTRG.20417.1	48.85	590.82	389.66
	MSTRG.31785.1	39.23	1034.24	347.41
Transcription factor MYC2 (MYC2)	MSTRG.16496.1	10.34	4.79	32
	MSTRG.4905.1	42.25	157.63	78.08
Mitogen-activated protein kinase 4 (MPK4)	MSTRG.830.2	59.38	26.18	47.95
Vegetative storage protein 2 (VSP2)	MSTRG.2523.1	31	2.05	7.33
	MSTRG.3467.1	15.46	0.42	2.09
**Ethylene**				
Ethylene-insenstive 3-like (EIL1)	MSTRG.4336.1	13.69	1.17	22.23
	MSTRG.3297.1	49.67	12.67	92.14
Ethylene-responsive transcription factor (ERF094)	MSTRG.15462.1	0.11	16.62	1.75

WRKY70, functioning as a positive regulator of SA-mediated defense while concurrently suppressing the JA response, was also a focus of our investigation. The balance between SA and JA pathways is essential for appropriate immune responses, and WRKY70 plays a key role in this regulatory network. Our transcriptomic analysis identified a transcript (MSTRG.8862.1), corresponding to WRKY70. This transcript exhibited significant upregulation at 6 hpi, reflecting the early activation of SA-mediated defense mechanisms. The subsequent decline in expression at 12 hpi could indicate a shift in the regulatory landscape of defense responses as the infection progresses, potentially involving other transcription factors or hormonal signals (Table 2, Additional File 4). Pathogenesis-related protein 1 (PR1) family members are renowned for their abundant production in plants upon encountering pathogenic threats. PR1 gene expression has previously served as a reliable marker for evaluating SA-mediated disease resistance [18]. Our transcriptome analysis identified two transcripts (MSTRG.17950.1 and MSTRG.18009.1) encoding PR1 genes. Both transcripts showed a pronounced upregulation at 6 hpi, consistent with the early phase of defense response against *P. capsici*. The observed decrease in expression at 12 hpi suggests a dynamic regulation of PR1 genes, potentially reflecting a transition to other defense mechanisms or a decrease in the initial response as the plant adapts to the ongoing infection (Table 2, Additional File 4). We also examined other key components of the SA signaling pathway, including GRX4 and TGA transcription factors. GRX4 (MSTRG.5776.1) is involved in redox regulation and can influence the redox status of signaling proteins, potentially affecting their activity. The upregulation of GRX4 in infected samples suggests its role in modulating the redox environment during the defense response. Conversely, TGA1 (MSTRG.1644.1) and TGA4 (MSTRG.4306.1), which are part of the TGA transcription factor family that works in concert with NPR1, showed downregulation in infected samples (Table 2, Additional File 4). This downregulation could indicate a complex regulatory interplay, where TGA factors might be less active or regulated differently in response to ongoing infection stages.

The early upregulation of NPR1, WRKY70, and PR1 transcripts at 6 hpi underscores the initial activation of the SA-mediated defense response in *P. nigrum*. This early response is crucial for mounting a rapid defense against pathogen invasion. The subsequent decline in transcript levels at 12 hpi highlights the dynamic nature of plant immune responses, where the initial activation of defense genes is followed by a phase of adaptation or modulation. The observed patterns of expression for GRX4 and TGA transcription factors further emphasize the complexity of the SA signaling network. The upregulation of GRX4 suggests a role in maintaining the redox balance during defense, while the downregulation of TGA factors points to potential shifts in transcriptional regulation as the infection evolves.

### Jasmonic acid signalling pathway

The jasmonic acid (JA) signaling pathway is crucial for regulating plant defense responses, particularly against necrotrophic pathogens. The major genes involved in JA signalling pathway are Coronatine Insensitive 1 (COI1), Jasmonate ZIM domain protein 1 (JAZ1), and Transcription factor MYC2 (MYC2). Each of these components plays a distinct role in orchestrating the plant response to pathogen attack. COI1, an essential F-box protein for all JA responses, plays a critical role in assembling the SCF (COI1) E3 ubiquitin ligase complex, which in turn, recruits Jasmonate ZIM-domain proteins for degradation via the 26 S proteasome [19]. In our study, we identified the COI1 transcript (MSTRG.25426.1), which showed a slight increase in expression in the infected samples (Table 2, Additional File 4). This increase suggests that COI1 may be involved in the early stages of JA signaling activation in response to *P. capsici* infection. However, the relatively small increase observed might also indicate that COI1 role is more nuanced or that other regulatory mechanisms are modulating its activity in conjunction with or independent of JA signaling.

JAZ1 is a member of the TIFY protein family and functions as a transcriptional repressor in the JA signaling pathway. These proteins are characterized by the TIF[F/Y]XG motif within a larger conserved region (∼28 amino acids) known as the ZIM (or TIFY) domain, which play essential roles in mediating JA responses, JAZ proteins inhibit the activity of MYC2 transcription factors, thereby repressing JA-responsive gene expression [20]. Our investigation identified two JAZ1 transcripts (MSTRG.20417.1 and MSTRG.31785.1) that exhibited higher expression at 6 hpi and decreased expression at 12 hpi (Table 2, Additional File 4). This pattern indicates that JAZ1 proteins might initially accumulate to repress JA responses, but their levels subsequently decrease, potentially due to the activation of JA signaling pathways and the degradation of JAZ1 proteins via the COI1-mediated ubiquitination process. This dynamic regulation underscores the temporal control of JAZ1 in modulating the JA response during different infection stages.

The third gene we studied is MYC2, a crucial transcription factor that interacts with JAZ proteins to initiate JA signaling. In the absence of JA, JAZ proteins inhibit MYC2 activity, thereby repressing the expression of JA-responsive genes. Upon JA perception, JAZ proteins are degraded, releasing MYC2 to activate defense gene expression [21]. Our study revealed two MYC2 transcripts (MSTRG.16496.1 and MSTRG.4905.1) (Table 2, Additional File 4). One MYC2 transcript (MSTRG.16496.1) exhibited increased expression at 12 hpi, while the other (MSTRG.4905.1) showed higher expression at 6 hpi compared to 12 hpi. This suggests that different MYC2 transcripts may have complementary roles at various stages of infection, with some possibly contributing to early defense responses and others playing a role in sustained or late-stage responses. The differential expression of MYC2 transcripts highlights the complexity of JA signaling and the potential for varied transcriptional responses to infection over time. Additionally, we studied MPK4, a mitogen-activated protein kinase involved in various signaling pathways, including those related to JA and SA. MPK4 positively influences GRX4 in the SA signaling pathway, suggesting a role in coordinating responses between these pathways. Conversely, MPK4 negatively regulates MYC2 in the JA signaling pathway, impacting JA-responsive gene expression. Notably, the transcript corresponding to MPK4 (MSTRG.830.2) exhibited consistent expression across all samples (Table 2, Additional File 4). This steady expression could indicate a fundamental role of MPK4 in modulating both SA and JA pathways during the infection process. VSP2 (Vegetative Storage Protein 2) is another well-characterized JA-responsive gene involved in the plant defense against pathogens. It is regulated by MYC2, and its expression typically reflects the activation of JA signaling. transcripts related to VSP2 (MSTRG.2523.1 and MSTRG.3467.1) were downregulated in infected samples (Table 2, Additional File 4). This downregulation could be attributed to several factors, such as a shift in the JA signaling dynamics, potential suppression of JA responses in the later stages of infection, or interplay with other signaling pathways that modulate VSP2 expression.

Overall, the analysis of the JA signaling pathway components in response to *P. capsici* infection provides insights into the dynamic regulation of defense responses. The increase in COI1 expression suggests its involvement in early JA signaling, while the fluctuating levels of JAZ1 and MYC2 transcripts indicate a complex interplay between repression and activation of JA responses. The consistent expression of MPK4 highlights its role in sustaining the JA signaling pathway, while the downregulation of VSP2 reflects possible shifts in JA signaling during infection.

### Ethylene signalling pathway

The ethylene (ET) signaling pathway is integral to plant defense responses, orchestrating a variety of processes from growth regulation to stress responses. In conjunction with other hormone pathways, particularly JA, ET signaling helps modulate the plant immune response to pathogens [6]. This interplay between pathways ensures a coordinated and effective defense strategy. EIL1 is a key transcription factor in the ET signaling pathway. It acts in conjunction with JAZs-MYC2 from the JA signalling pathway, highlighting the cross-talk between these two pathways [22]. In our investigation, we identified two transcripts (MSTRG.4336.1 and MSTRG.3297.1) encoding the EIL1 gene in the *P. nigrum* transcriptome. Both transcripts exhibited reduced expression at 6 hpi compared to the control but displayed increased expression at 12 hpi (Table 2, Additional File 4). This pattern suggests a potential role for EIL1 in early response suppression, possibly to mitigate initial stress responses, with subsequent activation indicating a role in sustained defense mechanisms. The reduction in EIL1 expression at 6 hpi could be part of an initial regulatory adjustment, allowing the plant to manage early infection stages. The increased expression at 12 hpi may reflect a transition to more robust defense responses, as the plant adapts to ongoing pathogen presence. The ET and JA pathways intersect in the transcriptional activation of Ethylene Response Factor 1 (ERF1), a gene encoding a transcription factor that regulates the expression of genes involved in pathogen responses to impede disease progression [23]. Our study revealed that the ERF1 transcript (MSTRG15462.1) exhibited expression in the 6 hpi infected sample and decreased expression at 12 hpi (Table 2, Additional File 4). This temporal regulation suggests that ERF1 is initially activated as part of an early defense response, but its role might shift or diminish as the infection progresses. The initial increase in ERF1 expression aligns with its role in kickstarting defense mechanisms, while the subsequent decrease could indicate a shift in regulatory priorities or a possible involvement in modulating the intensity of the response as the plant adapts to the pathogen. The findings from our study underscore the critical role of EIL1 and ERF1 in the ET signaling pathway, highlighting their temporal regulation during *P. capsici* infection.

**Fig. 2:**
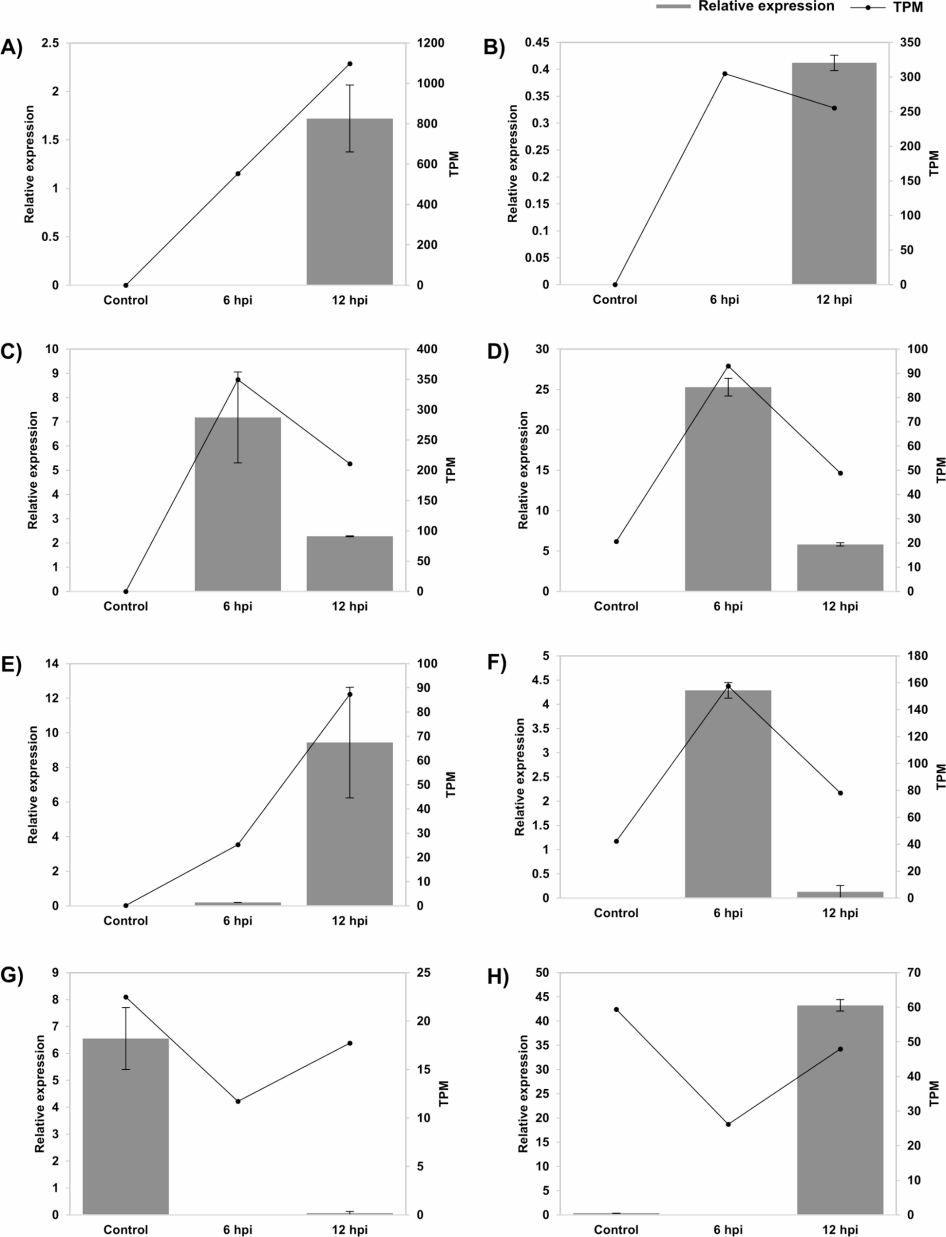
Validation of transcript expression using RT-qPCR analysis. selected transcripts from differentially expressed genes and hormone signalling pathways (**A**) Non-symbiotic hemoglobin 2 (MSTRG.298.1) (**B**) Putative norcoclaurine 6-O-methyltransferase (MSTRG.1147.1) (**C**) Corytuberine synthase (MSTRG.15304.2) (**D**) Fe2OG dioxygenase domain-containing protein (MSTRG.13487.1) (**E**) WRKy70 (MSTRG.8862.1) (**F**) MYC2 (MSTRG.4905.1) (**G**) TGA4 (MSTRG.4306.1) (**H**) MPK4 (MSTRG.830.2) were quantified in three samples using RT-qPCR. The grey-scale bars represent relative enzyme coding transcript expression in control, 6 hpi and 12 hpi by RT-qPCR analysis (left y-axis). The data represents mean values of three biological replicates for each sample. Black line represents TPM values of the transcripts by RNAseq (right y-axis)

To confirm these observed expression patterns from transcriptome analysis, we performed quantitative real-time polymerase chain reaction (RT-qPCR) analysis on selected transcripts (Fig. 2). We could observe a correlation between the expression levels derived from RT-qPCR and those determined by the transcriptome data for most of these transcripts in control, 6 hpi and 12 hpi. Overall, this study focused on the innate immunity of *P. nigrum* against *P. capsici*. Transcriptome analysis revealed the immune-related gene dynamics and allowed for the identification of differentially expressed transcripts. The significant activation of the SA and JA pathways early in infection, followed by the ET pathway involvement, shows *P. nigrum* complex defense mechanisms. The balance between these pathways is critical for optimizing resistance while minimizing potential trade-offs in growth and development. Our findings illustrate the complex regulatory networks involving SA, JA, and ET pathways in *P. nigrum* defense against *P. capsici*, emphasizing the dynamic interplay and temporal regulation of key signaling components. Moreover, hormone signalling pathways are known to have role in Systemic Acquired Resistance (SAR) and strategies have been developed by pathogens to manipulate plant hormonal pathways and modify the immune signaling for their own resistance enhancement in the host. This underscores the necessity for further in-depth studies in these lines.

## Conclusions

In this study, we investigated the molecular mechanisms underlying the innate immunity of *P. nigrum* against the oomycete pathogen *P. capsici*. By conducting transcriptome profiling and comparative transcriptomics, we identified DEGs in response to *P. capsici* infection. Our comprehensive transcriptome assembly, comprising 64,667 transcripts with high completeness, provided insight on the molecular processes underlying *P. nigrum* defensive mechanisms. Functional annotation of these transcripts revealed various functional categories and domains, allowing our understanding of the plant molecular repertoire. The differential gene expression analysis uncovered dynamic regulation of immune-related genes, with 4,714 transcripts at 6 hpi and 9,416 at 12 hpi showing significant differential expression. This dynamic regulation illustrates the complexities of the *P. nigrum* response to *P. capsici* and the plant ability to adapt and improve its defense mechanism. Our findings showed that the infection triggered a dynamic reprogramming of gene expression, with a significant increase in the expression of genes involved in the SA, JA, and ET signalling pathways. The SA signalling pathway is known to play a key role in the early defense response of plants against pathogens. We identified two transcripts of NPR1, a central regulator of the SA signalling pathway, which were significantly upregulated at 6 hpi and then declined at 12 hpi. Similarly, a transcript of WRKY70, a transcription factor positively regulating the SA response, exhibited a similar expression pattern. These results suggest that the SA signalling pathway is activated early in the infection process and then declines as the plant defense response progresses. The JA signalling pathway also plays a crucial role in defense response of plants against pathogens. We identified transcripts of COI1, JAZ1, and MYC2, key regulators of the JA signalling pathway. COI1 is an F-box protein that targets JAZ proteins for degradation, while MYC2 is a transcription factor that activates the JA response. Our results suggest that the JA signalling pathway is activated later in the infection process, following the decline of the SA signalling pathway. These sequential and complementary roles of these two signalling pathways may be significant and for understanding integrated disease resistance strategies. Furthermore, the ET signalling pathway was involved in the defense response, with dynamic expression of EIL1 and temporally regulated ERF1 during infection, elucidating their roles in disease progression. Overall, this study significantly contributes to our understanding of plant immunity and offers potential strategies for enhancing *P. nigrum* resistance to *P. capsici*. The molecular insights gained here provide a foundation for future research aimed at developing effective strategies for managing ‘quick wilt’ disease and ensuring the sustainability of *P. nigrum* cultivation.

## Materials and methods

### Plant, pathogen, and treatments

**Fig. 3:**
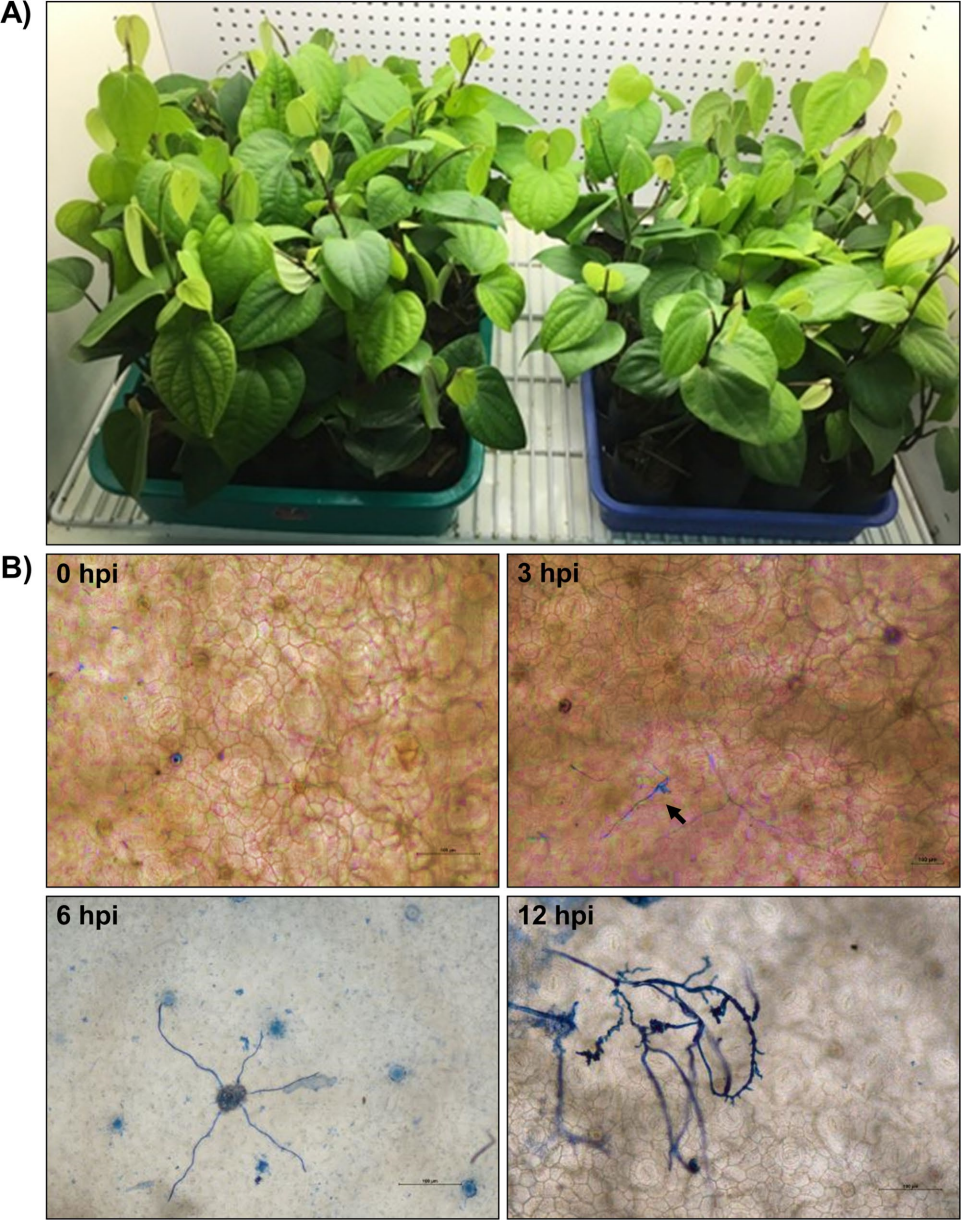
Morphology (**A**) Photograph of *P. nigrum* plantlets grown in controlled growth chambers condition (**B**) Trypan 657 blue staining of *P. capsici* shows the disease progression with early colonization of mycelia 658 at 0, 3, 6 and 12 hpi

Two-month-old plantlets of *Piper nigrum* L. var. Panniyur I, which shows high susceptibility to *P. capsici*, were maintained at the greenhouse facility of Rajiv Gandhi Centre for Biotechnology, Thiruvananthapuram, India, and used for all the studies (Fig. 3A). Infection assay experiments were carried out as described earlier [9, 24]. Briefly, *P. capsici* pv. *P. nigrum* grown axenically on potato dextrose agar medium (PDA) at 28 °C for four days was used for the infection assay experiment. The second leaf detached from at least ten *P. nigrum* plantlets were pinpricked once on the abaxial surface and further inoculated with the mycelial plugs of *P. capsici* at multiple sites and were placed in humid transparent plastic covers and incubated for 6 h and 12 h in a controlled growth chamber (Conviron, Canada) maintained at 28 °C and a photoperiod of 16 h and an RH of 70%. After incubation, the mycelial plugs removed from the infection site, and leaf discs of at least 5.0 mm diameter were harvested using a paper puncher from the inoculation sites and immediately frozen in liquid nitrogen before total RNA isolation. Transcriptome sequencing experiment was carried out on the three sets of RNA derived from each treatment, immediately frozen in liquid nitrogen, and stored at -80 °C until further use [9, 25]. For microscopic observation of the growth of *P. capsici*, we have utilized a trypan blue staining technique reported earlier [26]. The resulting leaf discs were observed under a light microscope (Leica Microsystems DM750) at a magnification of 40X (Fig. 3B).

### Total RNA isolation and Illumina sequencing

Total RNA was extracted from three different leaf disc pools using the RNeasy Plant mini kit (Qiagen) as per manufacturer recommendations. RNA isolated was treated with DNase I (Ambion, TX) to remove possible genomic contaminations. The RNA quantity was analyzed by spectrophotometry using Nanodrop (Thermo Scientific), and the quality was visually verified on 3% agarose gel. The total RNA integrity was assessed on an Agilent Bioanalyzer 2100 (Agilent Technologies, CA). Four micrograms of RNA with a minimum RIN value > 8 were used for cDNA library preparation according to the Illumina TruSeq RNA sample preparation kit (Illumina Inc., San Diego, CA, USA). Bioanalyzer plots were used at every step to assess mRNA quality, enrichment success, fragmentation, and final library sizes. Both Qubit (Invitrogen) and RT-qPCR were used for measuring the quantity of the library before sequencing. After the libraries were constructed, the cDNA was sequenced on Illumina HiSeq 2000 to obtain 100 bp paired-end data.

### *De novo* assembly and annotation of transcriptome data

Afte sequencing, Illumina RNA-Seq data was processed to generate FASTQ files. RNA-Seq data in FASTQ files were initially filtered to remove adaptor sequences using a fastqmcf tool (https://github.com/ExpressionAnalysis/ea-utils/blob/wiki/FastqMcf.md). We filtered out reads with an average quality score (Phred value) < 20 and a read length < 40 in any of the paired-end reads. The FASTQ sequence files were deposited and are available for public at NCBI under the Bioproject PRJNA318916. The reads were first aligned to a high-quality genome assembly of *P. nigrum* [27] using HISAT2 with default parameters [28]. StringTie2 was used to assemble the aligned reads for all samples into transcripts [29]. The transcripts from each sample were further merged using the StringTie2 merge function to create a common set of transcripts for all the samples. TransDecoder with a minimum threshold of 100 residues in length was used to predict unigenes (ORFs) from the transcripts. The host (*P. nigrum*) and pathogen (*P. capsici*) transcripts were classified corresponding to Viridiplantae and Stramenopiles (oomycetes) based queries. The assembled transcripts and predicted unigenes from the transcriptome were annotated further using BLASTX and BLASTP [30] searched against Uniprot Viridiplantae [31] database and HMMSCAN against Pfam database [32, 33]. Matches with a stringent cut-off of expected value (e-value) ≤ 1e-5 and similarity ≥ 40% were retained for further annotation. The quality of the transcriptome assembly further evaluated using BUSCO v5 [34] against Viridiplantae database.

### Differential gene expression analysis

The differential expression of transcripts were analysed using edgeR [35]. Initially, the featureCounts program [36] was utilized to quantify the transcripts in each sample. The resulting transcript counts were then utilized to calculate the fold change in expression and significance (P-value) of genes. DEApp [37] was employed to conduct a multi-factor experiment to analyse differential expression between pairs of groups. Genes exhibiting a fold change greater than 1.5 and a false discovery rate (FDR, Benjamini and Hochberg’s method) lower than 0.05 were identified as differentially expressed genes (DEGs). Volcano plots were generated to visualize the differential regulation, aiding in the identification of the differentially regulated genes.

### Identification of genes related hormone signalling pathways from transcriptome assembly

To investigate the genes associated with the SA, JA, and ET signalling pathways, we utilized the CAPS_protocol [38], a pipeline developed to identify enzymes coding transcripts from transcriptome. Clustal Omega [39] was employed for sequence alignment, and two iterations of jumpstart PSI-BLAST [40] were performed against the *P. nigrum* transcriptome assembly. The Clustal Omega alignment was used as input, with an E-value threshold of 1e^− 5^ was applied. Hits that exhibited a query coverage of at least 70% and a percentage identity of at least 40% were further analyzed. True hits were identified by aligning these hits with the query sequences and comparing functionally significant residues. MEGA software [41] was used, in conjunction with MUSCLE module [42], for sequence alignment to generate phylogenetic trees. The maximum likelihood method, with a bootstrap value of 1000, was employed to construct the phylogenetic trees.

### RNA isolation, cDNA synthesis, and transcript level expression analysis using RT-qPCR

The Nucleospin^®^ RNA plant kit (Macherey-Nagel), was used to extract the total RNA from leaf discs of control and infected leaf discs. One µg of total RNA from each sample was used for the first-strand cDNA synthesis using the PrimeScriptTM RT Reagent Kit (Perfect Real Time), according to the manufacturer’s instructions. The Primer3Plus software (https://primer3plus.com/cgi-bin/dev/primer3plus.cgi) was used to synthesize primer sequences for RT-qPCR analysis of the genes selected (Additional File 5). SYBR Premix Ex Taq II (Takara) was used in the RT-qPCR analysis using the QuantStudio 5 Real-Time PCR instrument (Applied Biosystems). 10 µl SYBR Green PCR Premix (Takara), 2 µl cDNA template, 0.4 µl ROX Reference Dye (50X), 0.8 µl each of the forward and reverse primers, and 6 µl of sterile, nuclease-free water were added to the 20 µl PCR volumes. 50 °C for two minutes, 95 °C for ten minutes, and 40 cycles of 15 s at 95 °C and 1 min at 60 °C were the conditions for the PCR. For the quantitative gene expression experiments, the comparative CT approach (2 − ΔΔCT method) was applied. The expression data was normalized using *P. nigrum* 18 S rRNA.

## Electronic supplementary material

Below is the link to the electronic supplementary material.

Supplementary Material 1

Supplementary Material 2

Supplementary Material 3

Supplementary Material 4

Supplementary Material 5

Supplementary Material 6

## Data Availability

Sequence data that support the findings of this study have been deposited at NCBI Bio project with the primary accession code PRJNA318916.

## References

[1] Anandaraj M, Sarma YR. Diseases of black pepper (Piper nigrum L) and their management. 1995.

[2] Balakrishnan R, Anandaraj M, Nambiar KKN, Sarma YR, Brahma RN, ESTIMATES ON THE EXTENT OF LOSS DUE TO QUICK WILT DISEASE OF BLACK PEPPER (PIPER NIGRUM L. IN CAUCUT DISTRICT OF KERALA. ) 1986.

[3] Gordo S, Pinheiro DG, Moreira ECO, Rodrigues SM, Poltronieri MC, de Lemos OF, et al. High-throughput sequencing of black pepper root transcriptome. BMC Plant Biol. 2012;12:1–9. 22984782 10.1186/1471-2229-12-168 PMC3487918

[4] Pieterse CMJ, der Does D, Zamioudis C, Leon-Reyes A, Van Wees SCM. Hormonal modulation of plant immunity. Annu Rev Cell Dev Biol. 2012;28:489–521. 22559264 10.1146/annurev-cellbio-092910-154055

[5] Wasternack C, Song S. Jasmonates: biosynthesis, metabolism, and signaling by proteins activating and repressing transcription. J Exp Bot. 2017;68:1303–21. 27940470 10.1093/jxb/erw443

[6] Broekaert WF, Delauré SL, De Bolle MFC, Cammue BPA. The role of ethylene in host-pathogen interactions. Annu Rev Phytopathol. 2006;44:393–416. 16602950 10.1146/annurev.phyto.44.070505.143440

[7] Rezzonico F, Rupp O, Fahrentrapp J. Pathogen recognition in compatible plant-microbe interactions. Sci Rep. 2017;7:6383. 28743967 10.1038/s41598-017-04792-5 PMC5526865

[8] Joy N, Asha S, Mallika V, Soniya EV. De novo transcriptome sequencing reveals a considerable bias in the incidence of simple sequence repeats towards the downstream of ‘pre-miRNAs’ of black pepper. PLoS ONE. 2013;8:e56694. 23469176 10.1371/journal.pone.0056694 PMC3587635

[9] Mahadevan C, Krishnan A, Saraswathy GG, Surendran A, Jaleel A, Sakuntala M. Transcriptome-assisted label-free quantitative proteomics analysis reveals novel insights into Piper nigrum—Phytophthora Capsici Phytopathosystem. Front Plant Sci. 2016;7:785. 27379110 10.3389/fpls.2016.00785 PMC4913111

[10] Indu M, Meera B, Sivakumar KC, Mahadevan C, Shafi KM, Nagarathnam B, et al. Priming’ protects Piper nigrum L. from Phytophthora capsici through reinforcement of phenylpropanoid pathway and possible enhancement of Piperine biosynthesis. Front Plant Sci. 2022;13.10.3389/fpls.2022.1072394. 10.3389/fpls.2022.1072394 PMC11908380 40093588

[11] Zhang H, Lang Z, Zhu J-K. Dynamics and function of DNA methylation in plants. Nat Rev Mol Cell Biol. 2018;19:489–506. 10.1038/s41580-018-0016-z. 29784956 10.1038/s41580-018-0016-z

[12] Cona A, Rea G, Angelini R, Federico R, Tavladoraki P. Functions of amine oxidases in plant development and defence. Trends Plant Sci. 2006;11:80–8. 16406305 10.1016/j.tplants.2005.12.009

[13] Kourelis J, Van Der Hoorn RAL. Defended to the nines: 25 years of resistance gene cloning identifies nine mechanisms for R protein function. Plant Cell. 2018;30:285–99. 29382771 10.1105/tpc.17.00579 PMC5868693

[14] Pires N, Dolan L. Origin and diversification of basic-helix-loop-helix proteins in plants. Mol Biol Evol. 2010;27:862–74. 19942615 10.1093/molbev/msp288 PMC2839125

[15] Bolton MD. Primary metabolism and plant defense—fuel for the fire. Mol plant-microbe Interact. 2009;22:487–97. 19348567 10.1094/MPMI-22-5-0487

[16] Yang J, Duan G, Li C, Liu L, Han G, Zhang Y, et al. The crosstalks between jasmonic acid and other plant hormone signaling highlight the involvement of jasmonic acid as a core component in plant response to biotic and abiotic stresses. Front Plant Sci. 2019;10:1349. 31681397 10.3389/fpls.2019.01349 PMC6813250

[17] Seyfferth C, Tsuda K. Salicylic acid signal transduction: the initiation of biosynthesis, perception and transcriptional reprogramming. Front Plant Sci. 2014;5. 10.3389/fpls.2014.00697. 10.3389/fpls.2014.00697 PMC4260477 25538725

[18] Breen S, Williams SJ, Outram M, Kobe B, Solomon PS. Emerging insights into the functions of pathogenesis-related protein 1. Trends Plant Sci. 2017;22:871–9. 10.1016/j.tplants.2017.06.013. 28743380 10.1016/j.tplants.2017.06.013

[19] Thines B, Katsir L, Melotto M, Niu Y, Mandaokar A, Liu G, et al. JAZ repressor proteins are targets of the SCFCOI1 complex during jasmonate signalling. Nature. 2007;448:661–5. 10.1038/nature05960. 17637677 10.1038/nature05960

[20] Chini A, Fonseca S, Fernandez G, Adie B, Chico JM, Lorenzo O, et al. The JAZ family of repressors is the missing link in jasmonate signalling. Nature. 2007;448:666–71. 17637675 10.1038/nature06006

[21] Fernández-Calvo P, Chini A, Fernández-Barbero G, Chico J-M, Gimenez-Ibanez S, Geerinck J, et al. The Arabidopsis bHLH transcription factors MYC3 and MYC4 are targets of JAZ repressors and act additively with MYC2 in the activation of jasmonate responses. Plant Cell. 2011;23:701–15. 21335373 10.1105/tpc.110.080788 PMC3077776

[22] Zhu Z, An F, Feng Y, Li P, Xue L, Jiang Z, et al. Derepression of ethylene-stabilized transcription factors (EIN3/EIL1) mediates jasmonate and ethylene signaling synergy in Arabidopsis. Proc Natl Acad Sci. 2011;108:12539–44. 21737749 10.1073/pnas.1103959108 PMC3145709

[23] Lorenzo O, Piqueras R, Sánchez-Serrano JJ, Solano R. ETHYLENE RESPONSE FACTOR1 integrates signals from ethylene and jasmonate pathways in plant defense. Plant Cell. 2003;15:165–78. 12509529 10.1105/tpc.007468 PMC143489

[24] Krishnan A, Mahadevan C, Mani T, Sakuntala M. Virus-induced gene silencing (VIGS) for elucidation of pathogen defense role of serine/threonine protein kinase in the non-model plant Piper Colubrinum Link. Plant Cell Tissue Organ Cult. 2015;122:269–83.

[25] Zuluaga AP, Vega-Arregu\’\in JC, Fei Z, Matas AJ, Patev S, Fry WE, et al. Analysis of the tomato leaf transcriptome during successive hemibiotrophic stages of a compatible interaction with the oomycete pathogen Phytophthora infestans. Mol Plant Pathol. 2016;17:42–54. 25808779 10.1111/mpp.12260 PMC6638369

[26] Chung C-L, Longfellow JM, Walsh EK, Kerdieh Z, Van Esbroeck G, Balint-Kurti P, et al. Resistance loci affecting distinct stages of fungal pathogenesis: use of introgression lines for QTL mapping and characterization in the maize-setosphaeria turcica pathosystem. BMC Plant Biol. 2010;10:1–25. 20529319 10.1186/1471-2229-10-103 PMC3017769

[27] Hu L, Xu Z, Wang M, Fan R, Yuan D, Wu B, et al. The chromosome-scale reference genome of black pepper provides insight into piperine biosynthesis. Nat Commun. 2019;10:4702. 10.1038/s41467-019-12607-6. 31619678 10.1038/s41467-019-12607-6 PMC6795880

[28] Kim D, Paggi JM, Park C, Bennett C, Salzberg SL. Graph-based genome alignment and genotyping with HISAT2 and HISAT-genotype. Nat Biotechnol. 2019;37:907–15. 10.1038/s41587-019-0201-4. 31375807 10.1038/s41587-019-0201-4 PMC7605509

[29] Kovaka S, Zimin AV, Pertea GM, Razaghi R, Salzberg SL, Pertea M. Transcriptome assembly from long-read RNA-seq alignments with StringTie2. Genome Biol. 2019;20:278. 10.1186/s13059-019-1910-1. 31842956 10.1186/s13059-019-1910-1 PMC6912988

[30] Altschul SF, Gish W, Miller W, Myers EW, Lipman DJ. Basic local alignment search tool. J Mol Biol. 1990;215:403–10. 10.1016/S0022-2836(05)80360-2. 2231712 10.1016/S0022-2836(05)80360-2

[31] Bateman A, UniProt. A worldwide hub of protein knowledge. Nucleic Acids Res. 2019;47:D506–15. 10.1093/nar/gky1049. 30395287 10.1093/nar/gky1049 PMC6323992

[32] Finn RD, Clements J, Eddy SR. HMMER web server: Interactive sequence similarity searching. Nucleic Acids Res. 2011;39 SUPPL. 2. 10.1093/nar/gkr367 PMC3125773 21593126

[33] Sonnhammer ELL, Eddy SR, Durbin R, Pfam. A comprehensive database of protein domain families based on seed alignments. Proteins Struct Funct Genet. 1997;28:405–20. doi: https://doi.org/10.1002/(SICI)1097-0134(199707)28:3>405::AID-PROT10>3.0.CO;2-L. 9223186 10.1002/(sici)1097-0134(199707)28:3<405::aid-prot10>3.0.co;2-l

[34] Nishimura O, Hara Y, Kuraku S. gVolante for standardizing completeness assessment of genome and transcriptome assemblies. Bioinformatics. 2017;33:3635–7. 10.1093/bioinformatics/btx445. 29036533 10.1093/bioinformatics/btx445 PMC5870689

[35] Robinson MD, McCarthy DJ, Smyth GK. edgeR: a Bioconductor package for differential expression analysis of digital gene expression data. Bioinformatics. 2010;26:139–40. 19910308 10.1093/bioinformatics/btp616 PMC2796818

[36] Liao Y, Smyth GK, Shi W. featureCounts: an efficient general purpose program for assigning sequence reads to genomic features. Bioinformatics. 2014;30:923–30. 24227677 10.1093/bioinformatics/btt656

[37] Li Y, Andrade J. DEApp: an interactive web interface for differential expression analysis of next generation sequence data. Source Code Biol Med. 2017;12:2. 10.1186/s13029-017-0063-4. 28174599 10.1186/s13029-017-0063-4 PMC5291987

[38] Joshi AG, Harini K, Meenakshi I, Shafi KM, Pasha SN, Mahita J, et al. A knowledge-driven protocol for prediction of proteins of interest with an emphasis on biosynthetic pathways. MethodsX. 2020;7:101053. 10.1016/j.mex.2020.101053. 33024710 10.1016/j.mex.2020.101053 PMC7528181

[39] Sievers F, Higgins DG. Clustal omega, accurate alignment of very large numbers of sequences. In: Russell DJ, editor. Methods in Molecular Biology. Totowa, NJ: Humana; 2014. pp. 105–16. 10.1007/978-1-62703-646-7_6. 10.1007/978-1-62703-646-7_6 24170397

[40] Karolewski Z, Fitt BDL, Latunde-Dada AO, Foster SJ, Todd AD, Downes K, et al. Gapped BLAST and PSI-BLAST: a new generation of protein database search programs. Plant Pathol. 2006;55:3389–402. 10.1111/j.1365-3059.1995.tb02715.x.

[41] Kumar S, Stecher G, Li M, Knyaz C, Tamura K. MEGA X: Molecular Evolutionary Genetics Analysis across Computing platforms. Mol Biol Evol. 2018;35:1547–9. 29722887 10.1093/molbev/msy096 PMC5967553

[42] Edgar RC. MUSCLE: a multiple sequence alignment method with reduced time and space complexity. BMC Bioinformatics. 2004;5:113. 10.1186/1471-2105-5-113. 15318951 10.1186/1471-2105-5-113 PMC517706

