# Supplementary material for: Transcriptional regulation of hormone signalling genes in black pepper in response to Phytophthora capsici
Source: BMC Genomics. 2024 Sep 30;25:910. doi: 10.1186/s12864-024-10802-4 (PMC11440725; doi:10.1186/s12864-024-10802-4)
Supplement: Supplementary file 1 — Supplementary Material 1 [file 12864_2024_10802_MOESM1_ESM.docx]

**Additional Files:**

**Additional File 1: Supplementary Table S1.** Result **s**ummary of the raw reads of Illumina sequencing.

**Additional File 2: Supplementary Table S2.** List of identified top 15 upregulated and downregulated transcripts from differential expression analysis.

**Additional File 3: Supplementary File S1.** Top 20 GO terms from three categories Biological Process (BP), Molecular Function (MF) and Cellular Component (CC) enriched in differentially expressed genes.

**Additional File 4: Supplementary File S2.** Functionally important residues mapping for transcripts encoding enzymes involved in hormone signalling pathways. The multiple sequence alignment was generated using known enzyme sequences from closely related species.

**Additional File 5: Supplementary Table S3.** Primers designed for selected transcripts.
