# Supplementary material for: Transcriptional regulation of hormone signalling genes in black pepper in response to Phytophthora capsici
Source: BMC Genomics. 2024 Sep 30;25:910. doi: 10.1186/s12864-024-10802-4 (PMC11440725; doi:10.1186/s12864-024-10802-4)

Supplementary File S1. Top 20 GO terms from three categories Biological Process (BP), Molecular Function (MF) and Cellular Component (CC) enriched in differentially expressed genes.

GO term enrichment in upregulated genes at 6 hpi

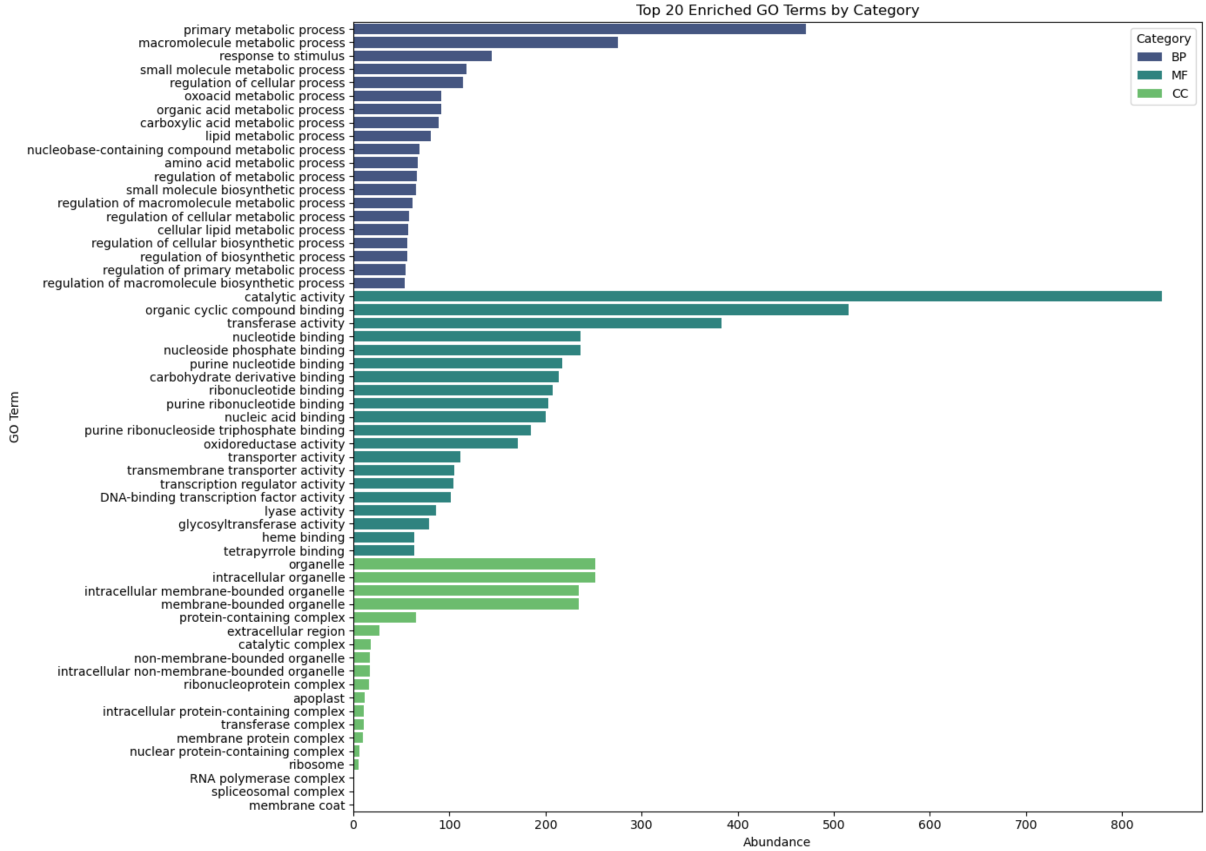

GO term enrichment in downregulated genes at 6 hpi

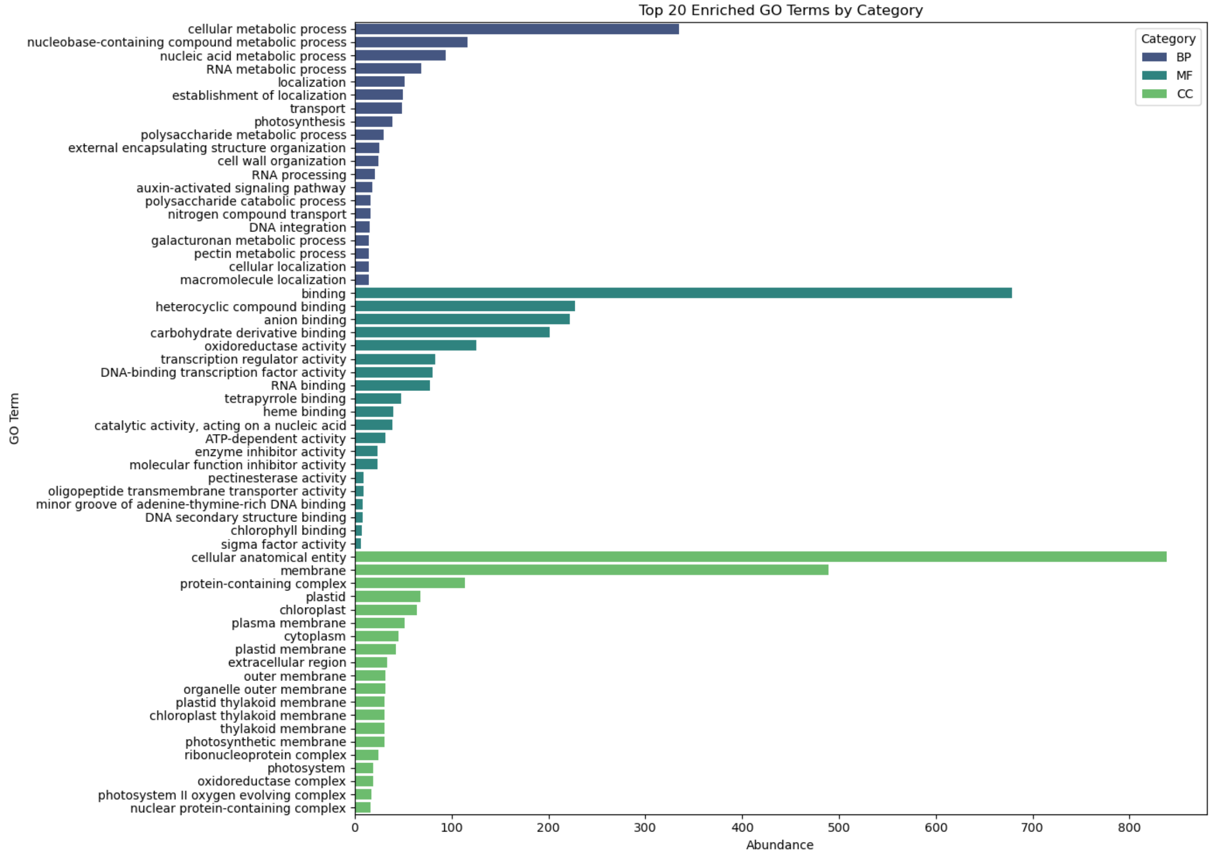

GO term enrichment in upregulated genes at 12 hpi

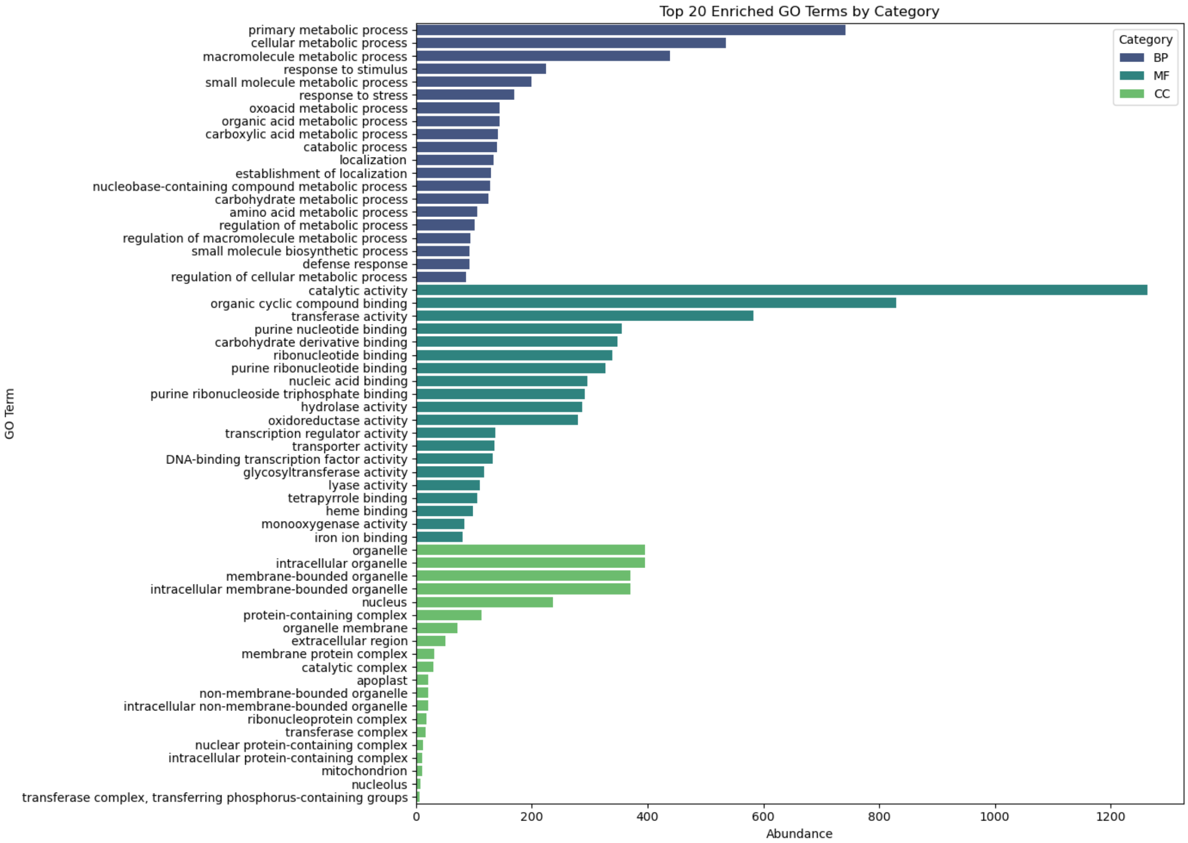

GO term enrichment in downregulated genes at 12 hpi

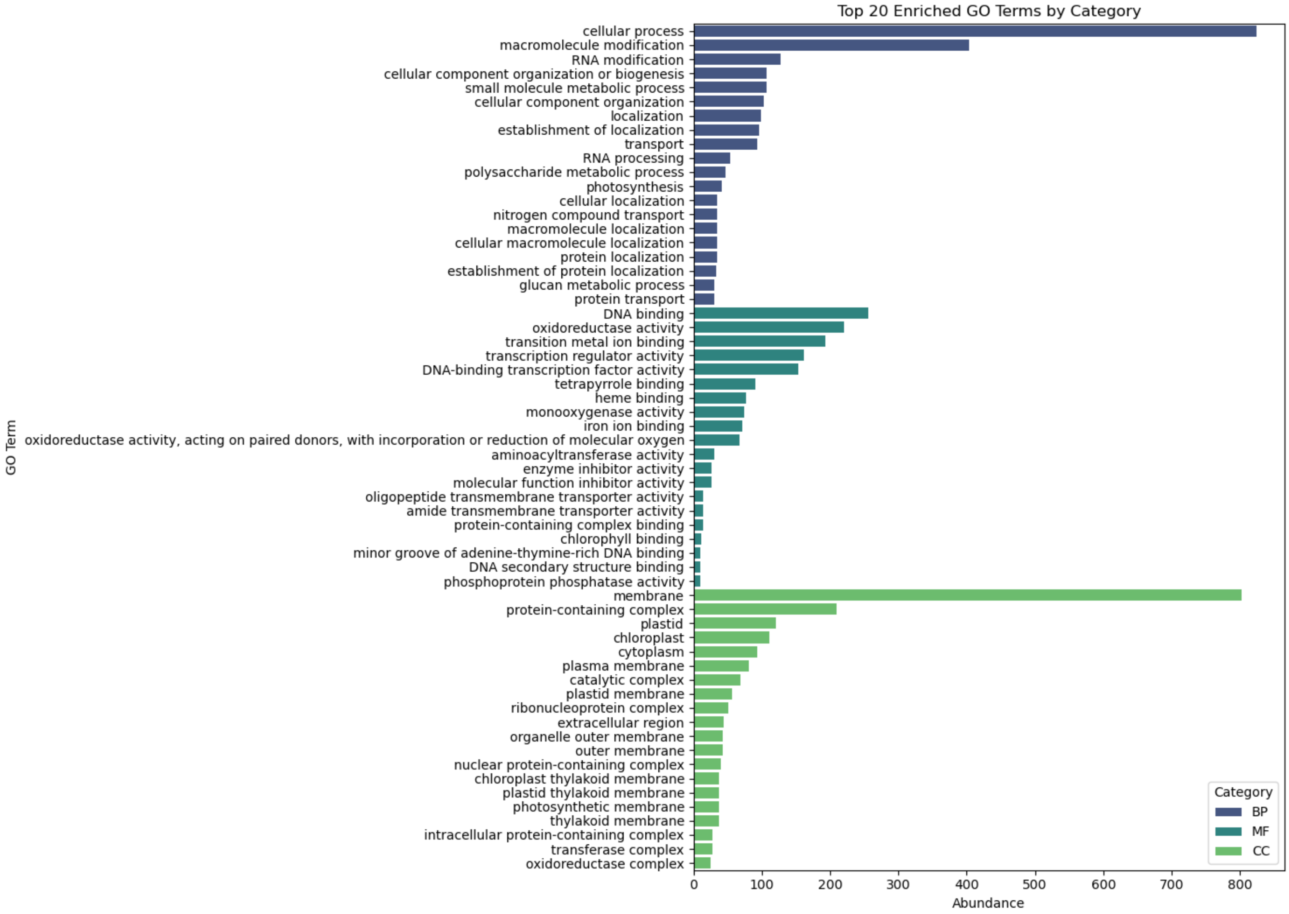

Supplement: Supplementary file 4 — Supplementary Material 4 [file 12864_2024_10802_MOESM4_ESM.pdf]
