# Supplementary material for: Transcriptional regulation of hormone signalling genes in black pepper in response to Phytophthora capsici
Source: BMC Genomics. 2024 Sep 30;25:910. doi: 10.1186/s12864-024-10802-4 (PMC11440725; doi:10.1186/s12864-024-10802-4)
Supplement: Supplementary file 5 — Supplementary Material 5 [file 12864_2024_10802_MOESM5_ESM.pdf]

Supplementary File S2. Functionally important residues mapping for transcripts encoding enzymes involved in hormone signalling pathways. The multiple sequence alignment was generated using known enzyme sequences from closely related species.

NPR1 (Non-expressor of pathogenesis-related genes 1)

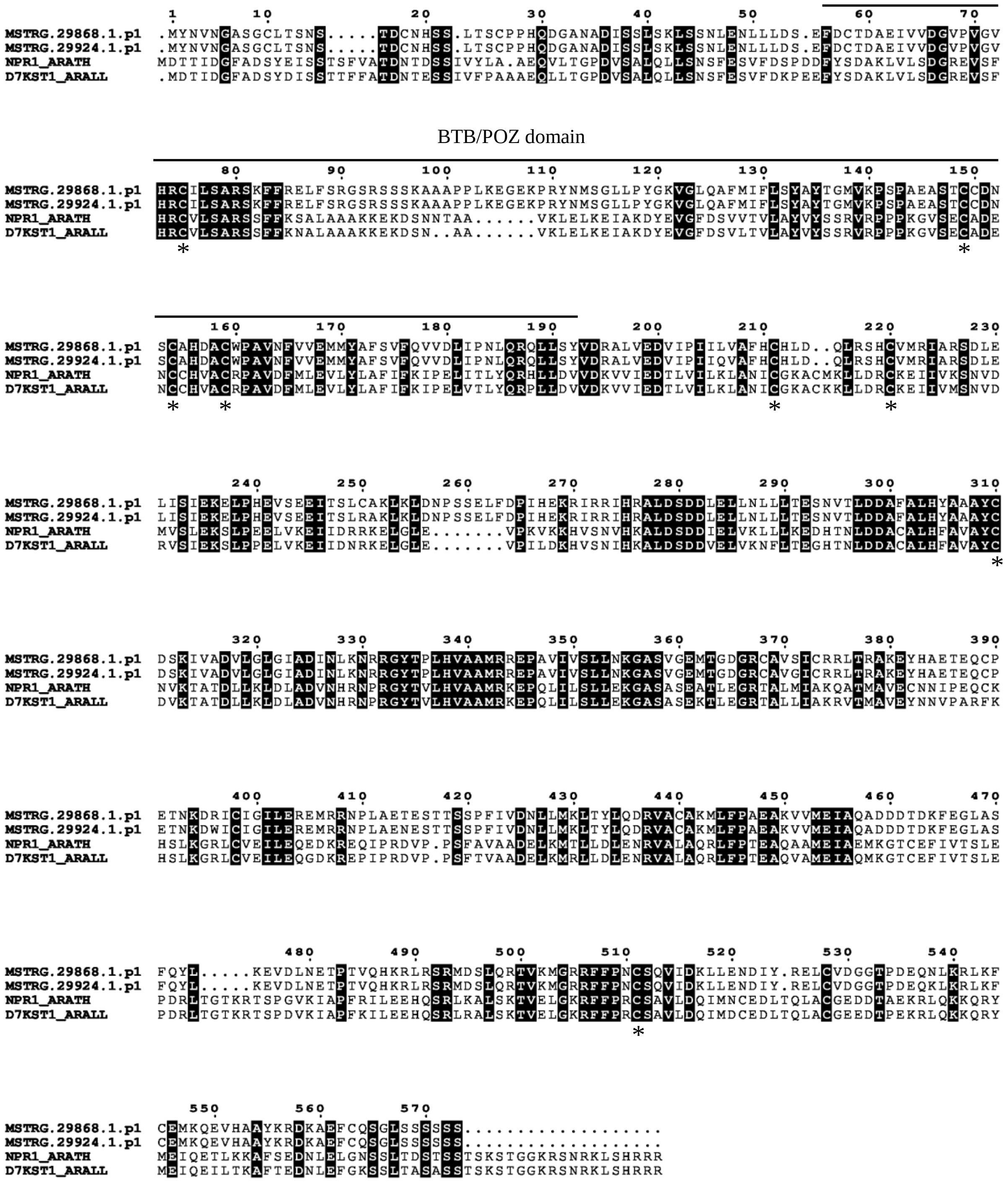

* Cysteine residues: involved in the formation of disulfide bonds

Citation: Sun L-M, Fang J-B, Zhang M, Qi X-J, Lin M-M and Chen J-Y (2020) Molecular Cloning and Functional Analysis of the NPR1 Homolog in Kiwifruit (Actinidia eriantha). Front. Plant Sci. 11:551201. doi: 10.3389/fpls.2020.551201

WRKY70 (WRKY transcription factor 70)

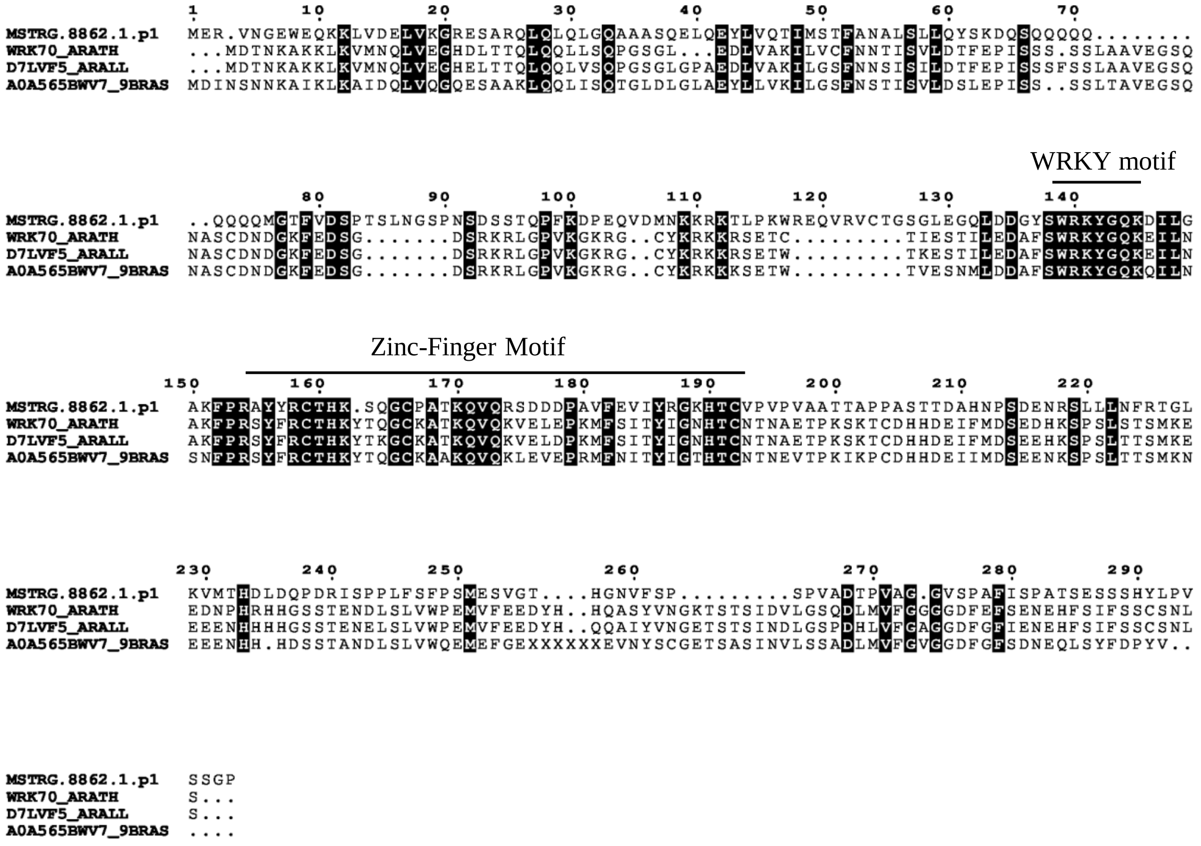

Citation: Sun S, Li X, Gao S, Nie N, Zhang H, Yang Y, He S, Liu Q, Zhai H. A Novel WRKY Transcription Factor from Ipomoea trifida, ItfWRKY70, Confers Drought Tolerance in Sweet Potato. International Journal of Molecular Sciences. 2022; 23(2):686. https://doi.org/10.3390/ijms23020686

Glutaredoxin-C9 (GRX4)

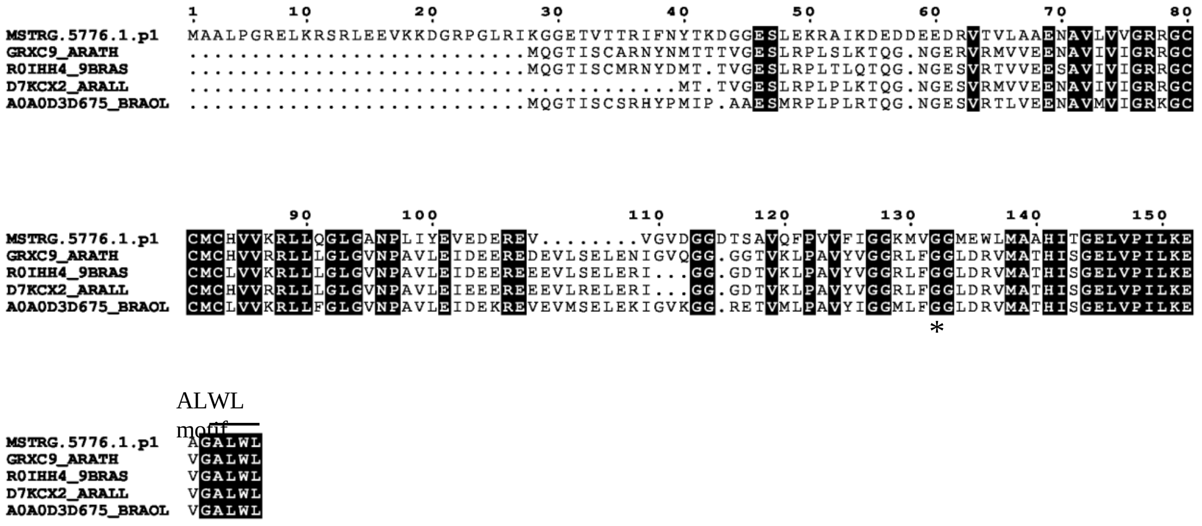

* Putative glutathione (GSH)-binding site

Citation: La Camera, S., L'Haridon, F., Astier, J., Zander, M., Abou-Mansour, E., Page, G., Thurow, C., Wendehenne, D., Gatz, C., Métraux, J.-P. and Lamotte, O. (2011), The glutaredoxin ATGRXS13 is required to facilitate Botrytis cinerea infection of Arabidopsis thaliana plants. The Plant Journal, 68: 507-519. https://doi.org/10.1111/j.1365-313X.2011.04706.x

Transcription factor (TGA1)

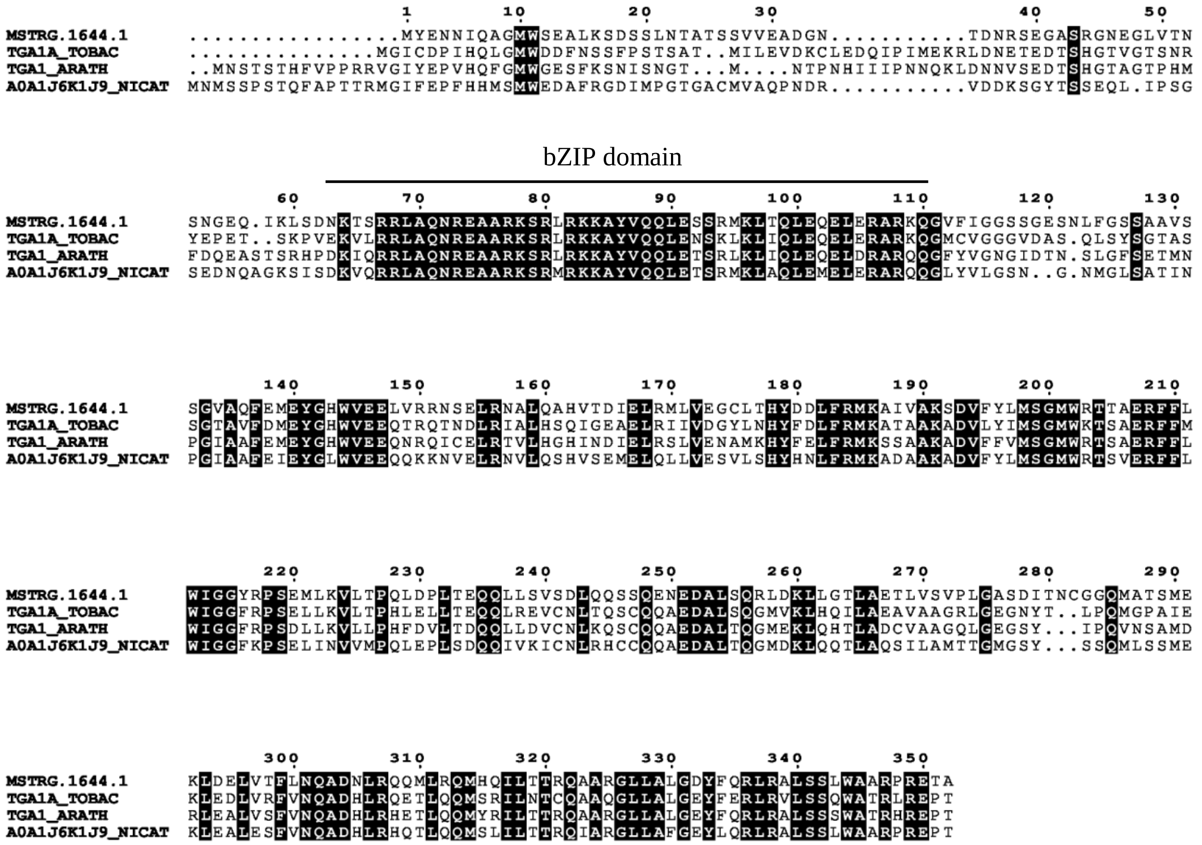

Citation: Tomaž Š, Gruden K and Coll A (2022) TGA transcription factors—Structural characteristics as basis for functional variability. Front. Plant Sci. 13:935819. doi: 10.3389/fpls.2022.935819

Transcription factor (TGA4)

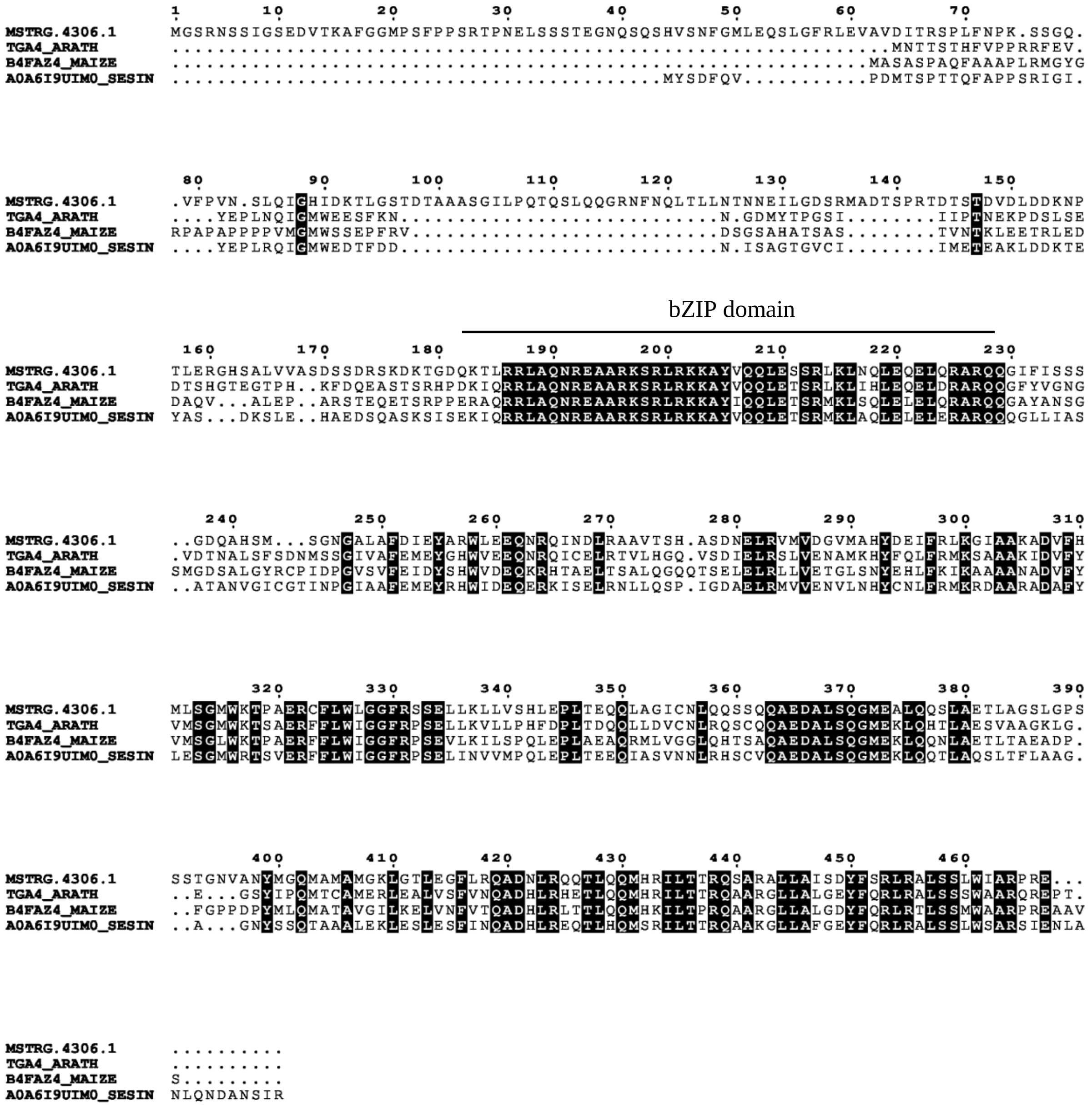

Citation: Tomaž Š, Gruden K and Coll A (2022) TGA transcription factors—Structural characteristics as basis for functional variability. Front. Plant Sci. 13:935819. doi: 10.3389/fpls.2022.935819

COI1 (Coronatine Insensitive 1)

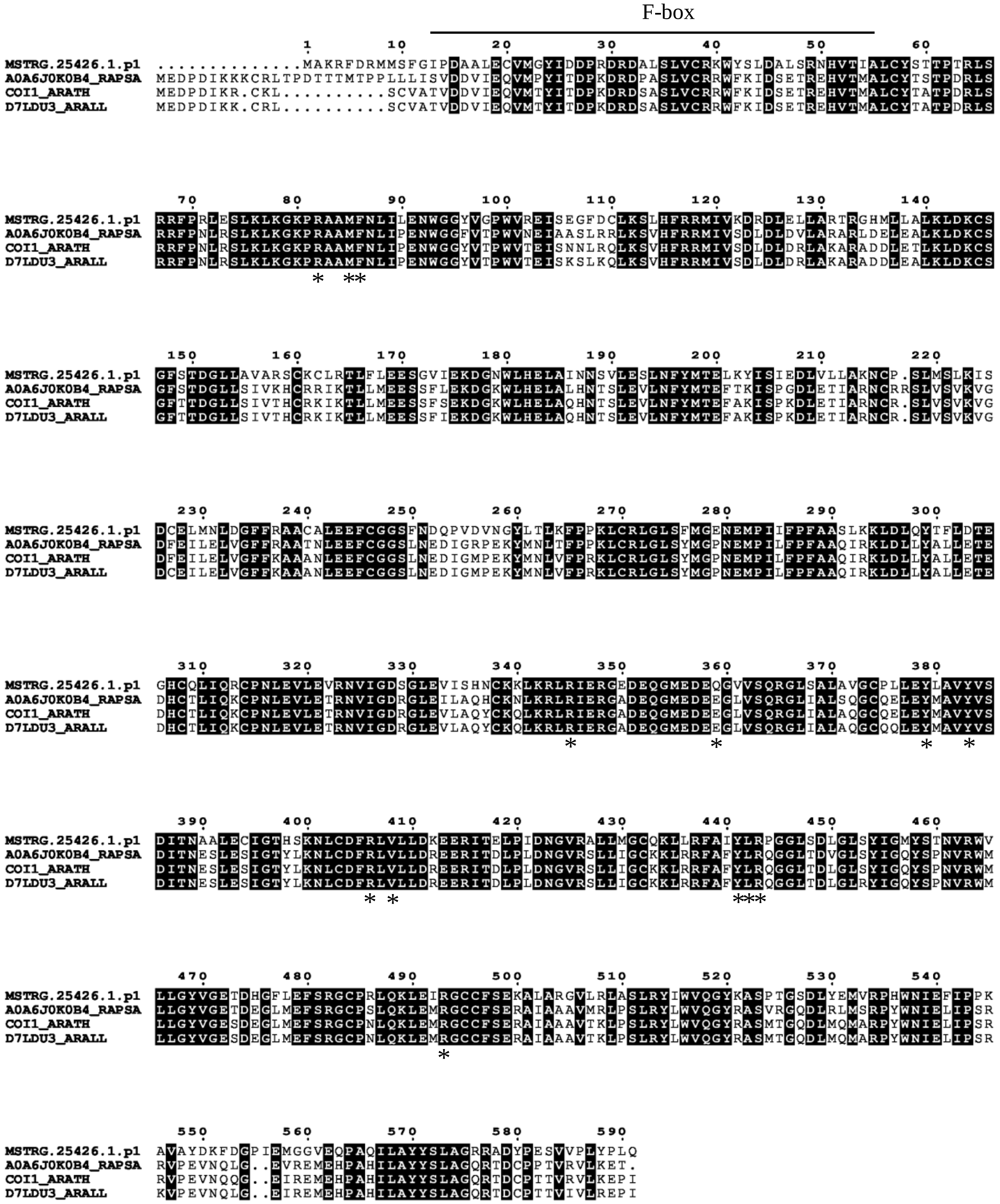

∗ Binding sites of coronatine/JA-Ile in the COI1-JAZcomplex

Citation: An, L., Ahmad, R.M., Ren, H. *et al.* Jasmonate Signal Receptor Gene Family *ZmCOIs* Restore Male Fertility and Defense Response of *Arabidopsis* mutant *coi1-1*. *J Plant Growth Regul* **38**, 479–493 (2019). https://doi.org/10.1007/s00344-018-9863-2

JAZ1 (Jasmonate ZIM domain protein 1)

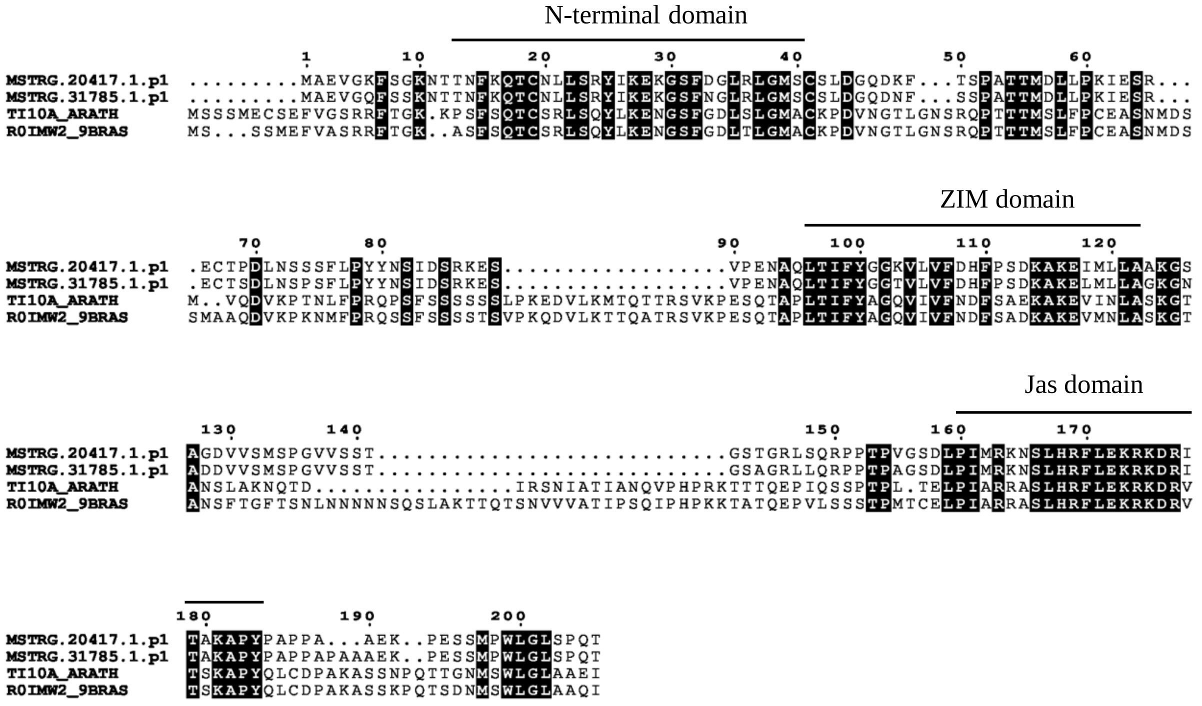

Citation: Zhu D, Li R, Liu X, Sun M, Wu J, Zhang N, et al. (2014) The Positive Regulatory Roles of the TIFY10 Proteins in Plant Responses to Alkaline Stress. PLoS ONE 9(11): e111984. https://doi.org/10.1371/journal.pone.0111984

MYC2 (Transcription factor MYC2)

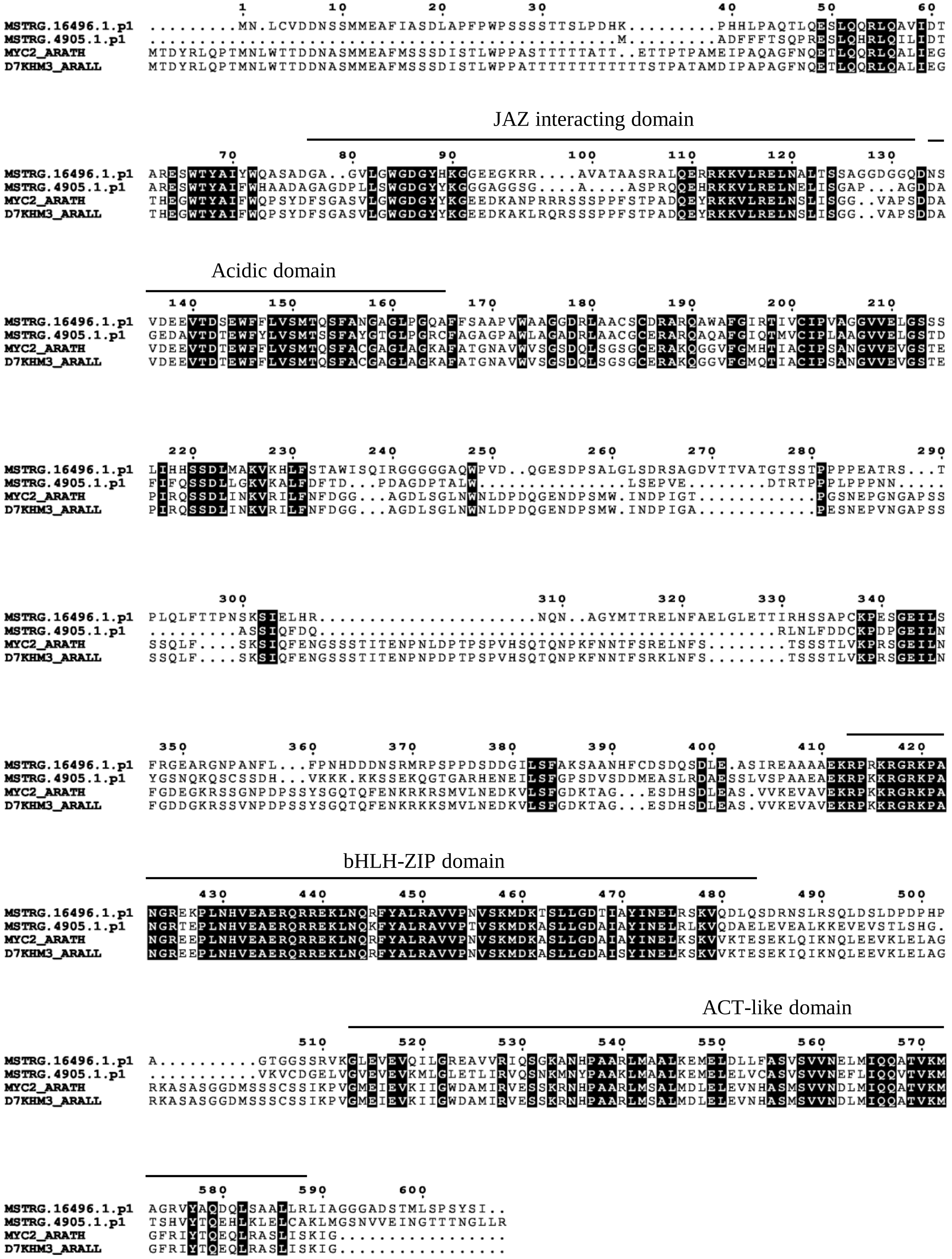

Citation: QiCui, XueGao, Lian-juanWang, and Gui-xiaJia. 2021. Ectopic expression of LhMYC2 increases susceptibility to Botrytis cinerea in Arabidopsis thaliana. Canadian Journal of Plant Science. 101(3): 328-340. https://doi.org/10.1139/cjps-2020-0047

Mitogen-activated protein kinase 4 (MPK4)

```
                  1       10        20        30        40        50        60        70        80
MSTRG.830.2.p1    MAGQPPDADMADAPPPPPPPQQPPGFDSIPALLSHGGRFIQYNIFGNIFEVTSKYKPPILPIGKGAYGIVCSALNSETGE
MPK4_ARATH        .........MSAE....SCFGSSGDQSSSKGVATHGGSYVQYNVYGNLFEVSRKYVPPLRPIGRGAYGIVCAATNSETGE
D7M4W5_ARALL      .........MSAE....TCFGSSGDQS.SKGLPTHGGSYVQYNVYGNLFEVSRKYVPPLRPIGRGAYGIVCAATNSETGE
V4LD72_EUTSA      .........MSAE....SCFGGSGDQS.TKGLPTHGGRYVQYNVYGNLFEVSRKYVPPLRPIGRGAYGIVCAATNSETGE

                          90       100       110       120       130       140       150       160
MSTRG.830.2.p1    QVAIKKITNAFDNKIDAKRTLREIKLLRHMDHENVVAIRDIIPPPLRDSFNDVYISYELMDTDLHQIIRSNQALSEEHCQ
MPK4_ARATH        EVAIKKIGNAFDNIIDAKRTLREIKLLKHMDHENVIAVKDIIKPPQRENFNDVYIVYELMDTDLHQIIRSNQPLTDDHCR
D7M4W5_ARALL      EVAIKKIGNAFDNIIDAKRTLREIKLLKHMDHENVIAVKDIIKPPQRENFNDVYIVYELMDTDLHQIIRSNQPLTDDHCR
V4LD72_EUTSA      EVAIKKIGNAFDNIIDAKRTLREIKLLKHMDHENVIAVKDIIRPPQRENFNDVYIVYELMDTDLHQIIRSNQPLTDDHCR

                         170       180       190       200       210       220       230       240
MSTRG.830.2.p1    YFLYQILRGLKYIHSANVLHRDLKPSNLLLNANCDLKICDFGLARITSETDFMTEYVVTRWYRAPELLLNSSEYSAAIDV
MPK4_ARATH        FFLYQLLRGLKYVHSANVLHRDLKPSNLLLNANCDLKLGDFGLARTKSETDFMTEYVVTRWYRAPELLLNCSEYTAAIDI
D7M4W5_ARALL      FFLYQLLRGLKYVHSANVLHRDLKPSNLLLNANCDLKLGDFGLARTKSETDFMTEYVVTRWYRAPELLLNCSEYTAAIDI
V4LD72_EUTSA      FFLYQLLRGLKYVHSANVLHRDLKPSNLLLNANCDLKLGDFGLARTKSETDFMTEYVVTRWYRAPELLLNCSEYTAAIDI
                                                                       ***

                         250       260       270       280       290       300       310
MSTRG.830.2.p1    WSVGCIFMELMDRKPLFPGRDHVHQLRLLMELIGTPSESDLGCVN.ENARRYIQQLPRHPRQSFKEKFPSVNSLAIDLVE
MPK4_ARATH        WSVGCILGETMTREPLFPGKDYVHQLRLITELIGSPDDSSLGFLRSDNARRYVRQLPQYPRQNFAARFPNMSAGAVDLLE
D7M4W5_ARALL      WSVGCILGETMTREPLFPGKDYVHQLRLITELIGSPDDSSLGFLRSDNARRYVRQLPQYPRQNFAARFPNMSAGAVDLLE
V4LD72_EUTSA      WSVGCILGETMTREPLFPGKDYVHQLRLITELIGSPDDSSLGFLRSDNARRYVRQLPQYPRQNFAARFPNMSAGAVDLLE

                320       330       340       350       360       370       380
MSTRG.830.2.p1    RMLTFDPRRRISVEDALAHPYLESLHDESDEPICMTPFSFDFEQHALSEEQMKELIYREALAFNPDYR.
MPK4_ARATH        KMLVFDPSRRITVDEALCHPYLAPLHDINEEPVCVRPFNFDFEQPTLTEENIKELIYRETVKFNPQDSV
D7M4W5_ARALL      KMLVFDPSRRITVDEALCHPYLAPLHDINEEPVCVRPFNFDFEQPTLTEENIKELIYRETVKFNPQDSV
V4LD72_EUTSA      KMLVFDPSRRITVDEALCHPYLAPLHDINEEPVCVRPFNFDFEQPSLTEENIKELIYRETVKFNPQDSQ
```

* Highly conserved T-X-Y motif in the activation loop

Citation: Siodmak, A., Shahul Hameed, U.F., Rayapuram, N., Völz, R., Boudsocq, M., Alharbi, S., Alhoraibi, H., Lee, Y.-H., Blilou, I., Arold, S.T. and Hirt, H. (2023), Essential role of the CD docking motif of MPK4 in plant immunity, growth, and development. New Phytol, 239: 1112-1126. https://doi.org/10.1111/nph.18989

Vegetative storage protein 2 (VSP2)

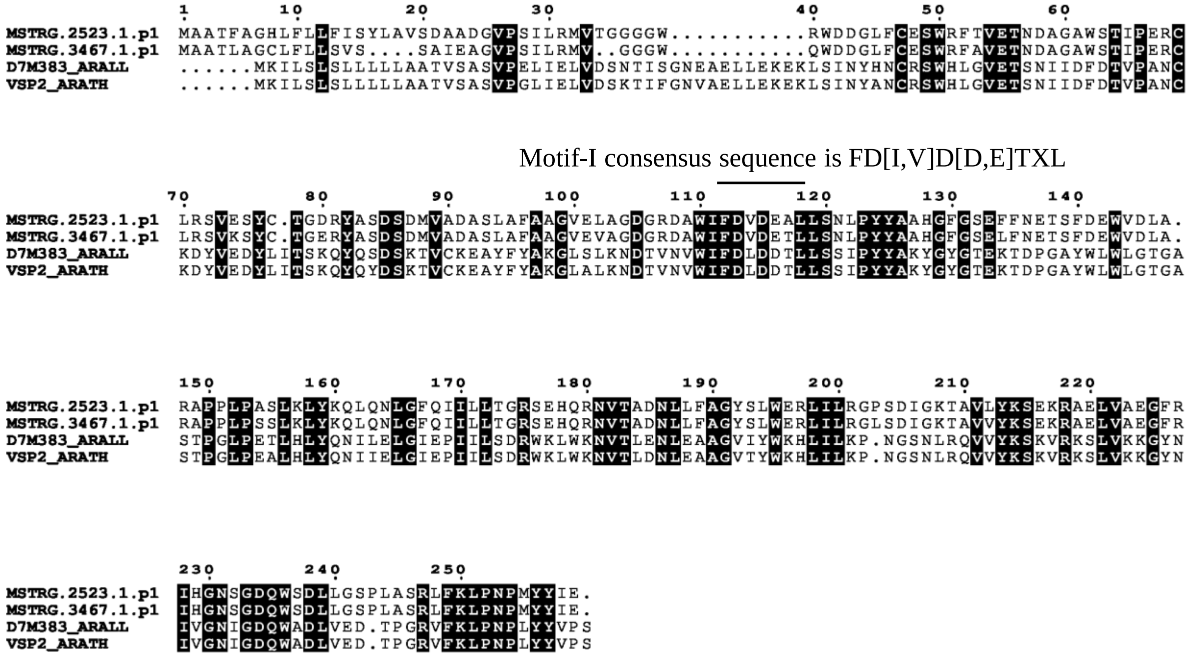

Citation: Leelapon, O., Sarath, G. & Staswick, P.E. A single amino acid substitution in soybean VSPα increases its acid phosphatase activity nearly 20-fold. Planta 219, 1071–1079 (2004). https://doi.org/10.1007/s00425-004-1294-6

EIL1/EIN3 (Ethylene Insensitive 3-like 1 / Ethylene Insensitive 3)

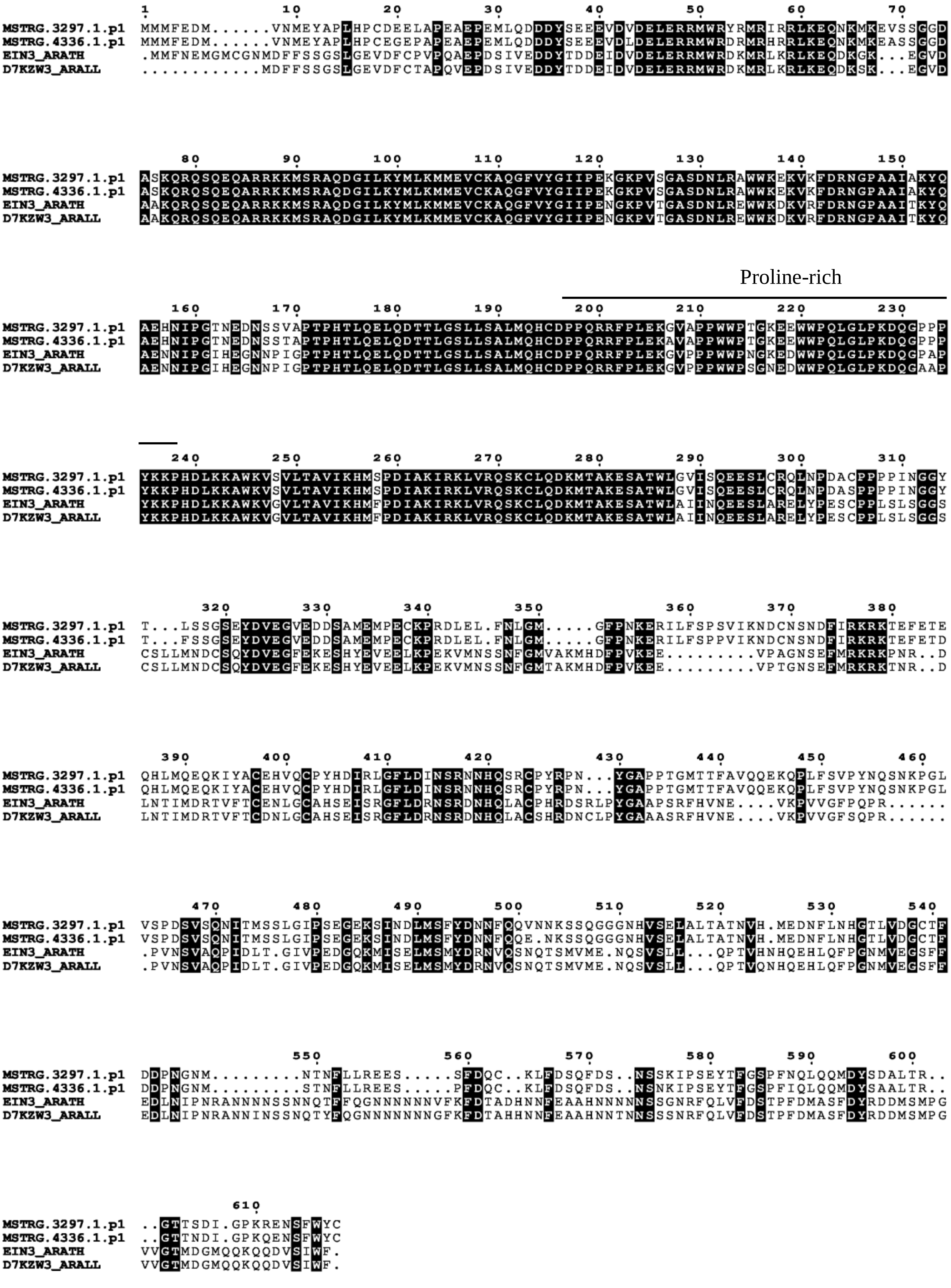

Citation: Song J, Zhu C, Zhang X, Wen X, Liu L, Peng J, et al. (2015) Biochemical and Structural Insights into the Mechanism of DNA Recognition by Arabidopsis ETHYLENE INSENSITIVE3. PLoS ONE 10(9): e0137439. https://doi.org/10.1371/journal.pone.0137439

ERF1 (Ethylene-responsive transcription factor 1)

```
MSTRG.15462.1.p1  ...MEMGSQSSALTAESSGSSSPESGSWELGIMINGADYKAARQELLPFNENDSEEMMLFEMLAEAGSREVVCMGSSVKE
ERF92_ARATH       MDPFLIQSPFSGFS..........PEYSIGSSPDSFSSSSSNNYSLPFNENDSEEMFLYGLIEQSTQQTYIDSDS..QD
D7L395_ARALL      MDPSFLQSPLSSFS..........PEYSTGSSPESFSSSSSNNYSLPFNENDSEEMFLYGLIKQSTQRNFIESNL..QD

                                                      ERF domain
MSTRG.15462.1.p1  EEIDSSAKTGAGAGELEEKSYRGVRKRPWGKFAAEIRDSTRRGIRVWLGTFDSAEAAALAYDQAAFAMRGSMAVLNFPEE
ERF92_ARATH       LP......IKSVSSRKSEKSYRGVRRRPWGKFAAEIRDSTRNGIRVWLGTFESAEEAALAYDQAAFSMRGSSAILNFSAE
D7L395_ARALL      LP......VKSVSSRNSEKSYRGVRRRPWGKFAAEIRDSTRNGIRVWLGTFESAEEAALAYDQAAFSMRGSSAILNFSPE

MSTRG.15462.1.p1  RVRESLREMAWSGSVASAGEEWCSPVVALKRRHSMKRKSSSSSSGKKKGKGEVVIRRESVVEFVDLGNEYLEQLLSSC..
ERF92_ARATH       RVQESLSEIKYT......YEDGCSPVVALKRKHSMRRRMTNKKTK.DSDFDHRSVKLDNVVVFEDLGEQYLEELLGSSEN
D7L395_ARALL      RVQESLSEMKYT......YEDGCSPVVALKRKHSMRRRKTNKKTK.DSDFDHRSVKLDNVVVFEDLGEQYLEELLGSSEN

MSTRG.15462.1.p1  ....
ERF92_ARATH       SGTW
D7L395_ARALL      SGTW
```

Citation: Susan Y. Fujimoto, Masaru Ohta, Akemi Usui, Hideaki Shinshi, Masaru Ohme-Takagi, Arabidopsis Ethylene-Responsive Element Binding Factors Act as Transcriptional Activators or Repressors of GCC Box–Mediated Gene Expression, The Plant Cell, Volume 12, Issue 3, March 2000, Pages 393–404, https://doi.org/10.1105/tpc.12.3.393
